# Metabolic organization of macaque visual cortex reflects visual field topography and perceptual specialization

**DOI:** 10.1371/journal.pbio.3003847

**Published:** 2026-06-08

**Authors:** Hiroki Oishi, Vladimir K. Berezovskii, Margaret S. Livingstone, Kevin S. Weiner, Michael J. Arcaro

**Affiliations:** 1 Department of Psychology, University of California, Berkeley, California, United States of America; 2 Division of Sensory and Cognitive Brain Mapping, Department of System Neuroscience, National Institute for Physiological Sciences, Okazaki, Aichi, Japan; 3 Graduate Institute for Advanced Studies, SOKENDAI, Hayama, Kanagawa, Japan; 4 Core for Spin Life Sciences, Okazaki Collaborative Platform, National Institutes of Natural Sciences, Okazaki, Aichi, Japan; 5 Department of Neurobiology, Harvard Medical School, Boston, Massachusetts, United States of America; 6 Department of Neuroscience, University of California, Berkeley, California, United States of America; 7 Helen Wills Neuroscience Institute, University of California, Berkeley, California, United States of America; 8 Department of Psychology, University of Pennsylvania, Philadelphia, Pennsylvania, United States of America; Massachusetts Institute of Technology, UNITED STATES OF AMERICA

## Abstract

Neural activity depends on energy metabolism, yet the extent to which regional variation in cortical metabolic architecture reflects the functional and perceptual demands of visual processing remains unclear. In the primate visual system, retinotopic eccentricity, the topographic mapping of visual space relative to gaze, provides a large-scale organizational axis along which spatial resolution and selectivity for behaviorally relevant visual categories vary systematically. Here, we tested whether cortical metabolic architecture reflects this axis by aligning *in vivo* fMRI maps of eccentricity and visual category selectivity with *ex vivo* cytochrome oxidase (CO) histology, a marker of oxidative metabolism, in macaque visual cortex. We found that the middle lateral (ML) face-selective region, which is biased toward central vision, exhibited higher CO intensity than the lateral place patch (LPP), a scene-selective region biased toward peripheral vision. More broadly, CO intensity covaried with eccentricity within both ML and LPP and across occipitotemporal visual cortex, though eccentricity only partially accounted for the elevated CO in ML. These findings reveal a close correspondence between cortical metabolic architecture and retinotopic organization, suggesting that the distribution of cortical metabolic resources is shaped by both visual field organization and the processing demands of perceptual specialization.

## Abstract 2

What factors can explain the brain’s energy usage patterns? This study shows that the distribution of a key metabolic marker in the primate visual system is not random but linked with functional responses, suggesting that cortical resource distribution is shaped by the processing demands of visual perception.

## Introduction

Neural activity underlying perception, cognition, and behavior is sustained by cortical energy metabolism. Given the brain’s high energetic demands [1], identifying the principles that govern how metabolic resources are distributed across cortical systems is central to understanding the biological constraints on neural computation. Recent studies in humans and nonhuman primates have revealed that cortical metabolic architecture is not spatially uniform across large scales [2,3]. However, the extent to which regional variation reflects functional organization remains unclear, particularly for large-scale systems that support behaviorally relevant computations. We hypothesize that cortical metabolic architecture is shaped by the processing demands of functionally specialized systems.

Energy production in neurons is primarily mediated by mitochondria through oxidative phosphorylation (OXPHOS) complexes located in the inner mitochondrial membrane. Cytochrome oxidase (CO), which catalyzes the terminal step of OXPHOS, has long been used as a histochemical marker of sustained metabolic demand driven largely by synaptic processing. Several studies have demonstrated that CO staining changes transsynaptically following prolonged alterations in functional input and is preferentially associated with dendritic and postsynaptic metabolic demands [4,5]. These findings indicate that regional differences in CO primarily reflect stable differences in metabolic architecture rather than transient stimulus-evoked activity.

CO histochemistry has long served as a key method for mapping cortical metabolic architecture, revealing discrete functional compartments across sensory systems [6]. Variations in CO intensity reflect differences in metabolic demand that have been linked to neural activity in early sensory cortices, such as CO-rich “blobs” in primary visual area V1 [7,8], barrels in somatosensory cortex [4], and tonotopically organized modules in auditory cortex [9]. These modules often coincide with high spontaneous firing rates and high metabolic demand of local circuits that are specialized for high-acuity or precision-tuned processing. Prominent staining in intermediate layers of primary cortices, such as layer IVc of V1 or layer IV of S1, also reflects the high metabolic costs of dense thalamic input processing. While this body of work has established the utility of CO staining for revealing functional subdivisions at the mesoscale, particularly in early sensory areas, much less is known about how metabolic architecture is distributed across cortical regions and hierarchies. In particular, the relationship between early metabolic specialization and downstream stages of processing, and whether large-scale functional systems exhibit corresponding patterns of energy metabolism, remains largely unexplored.

Inferotemporal (IT) cortex offers a strong test case for addressing this question. IT cortex contains spatially organized domains associated with distinct perceptual functions. A well-studied example is the contrast between face- and scene-selective regions. Face-selective areas emphasize fine-grained analysis of local features within central vision, a pattern well suited to identity recognition and expression decoding. In contrast, scene-selective regions are more sensitive to global configuration and spatial layout of the environment, integrating information across the visual periphery to support navigation and context perception [10–12]. These areas are not randomly distributed but instead align along a continuous topographic axis of retinotopic eccentricity, the mapping of visual space relative to gaze, that extends from the early visual cortex through IT [10,13,14]. This axis provides a consistent large-scale scaffold for functional specialization in the visual system and presents a principled framework for testing whether metabolic architecture reflects retinotopic organization.

Here, we investigated whether cortical metabolic architecture reflects large-scale functional organization in the primate visual system, focusing on retinotopic eccentricity and visual category selectivity. We combined *in vivo* fMRI mapping of eccentricity and category selectivity with *ex vivo* CO histology in macaque visual cortex. We compared CO intensity between two functionally and retinotopically distinct regions in IT: the face-selective patch ML, which is biased toward central visual input, and the scene-selective patch LPP, which is biased toward peripheral visual input. We then tested whether CO intensity covaried with eccentricity within these regions and across occipitotemporal visual cortex. CO intensity was higher in ML than in LPP and covaried with eccentricity both locally and across occipitotemporal visual cortex, revealing a metabolic gradient aligned with visual field organization. Notably, ML exhibited elevated CO levels beyond what could be explained by eccentricity alone, suggesting region-specific metabolic demands associated with face-selective cortex. These findings suggest that visual cortical metabolic architecture is shaped by both the spatial structure of visual sampling and the perceptual specializations that this sampling supports.

## Results

### CO architecture differs between face- and scene-selective regions

**Fig 1:**
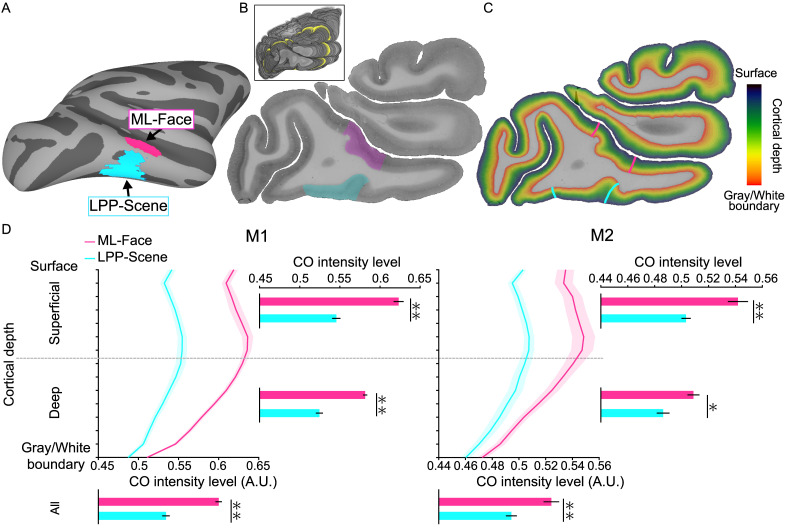
Cytochrome oxidase (CO) architecture differs between face and place patches. **(A)** A cortical surface of the right hemisphere, illustrating the anatomical locations of face-selective patch ML (magenta) and scene-selective patch LPP (cyan) from probabilistic maps (*n* = 6 for ML, *n* = 9 for LPP). **(B)** ML and LPP localized in a representative histological section stained for CO. ML and LPP are colored in translucent magenta and cyan, respectively. The inset image shows the 3D histological volume created by coregistering serial sections. The yellow-highlighted section corresponds to the representative section. ML and LPP were projected onto histological sections by aligning the 3D anatomical MRI to the histological volume. **(C)** Cortical depth maps. Colored lines illustrate the boundaries of ML and LPP. **(D)** Cortical depth profiles of CO intensity are shown for ML and LPP in the right hemispheres of monkey M1 (left) and M2 (right). Curves represent average CO intensity, with shading indicating ±1 SEM across histological sections. Bar plots to the right of each depth profile show mean CO intensity for superficial (top seven bins) and deep (bottom eight bins) portions of cortex. Mean CO intensity across the full cortical depth is shown below each profile. Asterisks indicate significant differences between ML and LPP (* *p* < 0.01, ** *p* < 0.001, FDR-corrected). Higher CO intensities indicate darker staining. The statistics of all comparisons are shown in S1 Table. The data underlying this Figure can be found in https://osf.io/gjbmd.

To assess metabolic architecture in the visual cortex, we leveraged a multimodal dataset combining *in vivo* fMRI and *ex vivo* histology from two macaque monkeys (M1 and M2). This dataset was previously used to examine variation in CO intensity across fMRI-defined face-selective patches in IT cortex, focusing on comparisons within a functionally coherent network [2]. In the present study, we extended this approach to test whether CO variation also reflects differences across functionally distinct networks within IT. Specifically, we compared CO intensity between two fMRI-defined regions with divergent category selectivity (Fig 1A). One region, the middle lateral (ML) face patch, was defined by strong neural responses to images of faces and weak responses to images of objects and scenes. The other region, the lateral place patch (LPP), responded strongly to scenes and weakly to images of objects and faces. Among the face-selective regions, we focused on ML because it is located at a similar anterior–posterior position as LPP, enabling a direct comparison while minimizing potential bias from the CO gradient that varies along this axis of IT cortex [2]. Comparisons involving more anterior face patches would be confounded by this gradient, making it difficult to attribute differences in CO intensity to category selectivity rather than anatomical position.

As described previously [2], cortical histological sections were reconstructed into 3D volumes by stacking and aligning adjacent 50-μm sagittal sections stained for CO (Fig 1B). Functional regions of interest (ROIs) were defined using probabilistic maps of category selectivity from fMRI, with the locations of ML and LPP corresponding to those reported across laboratories [11,12,15–17]. The probabilistic maps were aligned to the histological volume through intermediate registration to a high-resolution anatomical MRI (see Materials and methods). To characterize the distribution of CO across cortical depth, each section was segmented into 15 equal-volume bins at varying depths parallel to the cortical surface (Fig 1C). While these bins do not correspond to individual architectonic layers, they span the full thickness of the gray matter, allowing depth-resolved quantification of staining intensity.

To test whether category-selective regions differ in metabolic architecture, we compared the CO depth profiles between ML and LPP. In both regions, CO intensity increased from superficial to middle depths and then decreased toward deeper cortex, with peak staining in relatively superficial portions (Fig 1D). Although the overall depth profile was similar between regions, CO staining appeared consistently stronger in ML than in LPP across much of the cortical depth. We therefore asked whether this regional difference reflected a broad shift in CO intensity across depth or was restricted to a narrower depth range. To do so, we compared CO intensity between ML and LPP after averaging across the full cortical depth and separately within superficial and deep portions of cortex, defined as the top seven and bottom eight bins, respectively. ML exhibited significantly higher CO intensity than LPP in all three comparisons (all ts ≥ 2.948, all ps < 0.001 except for the deep portion in M2, where *p* = 0.006, false discovery rate (FDR)-corrected; Fig 1D and S1 Table). These findings indicate that face- and scene-selective regions in IT differ in metabolic staining intensity, and that this difference is evident across both superficial and deep portions of cortex.

### CO intensity correlates with eccentricity in both face- and scene-selective regions

The observed differences in CO intensity between ML and LPP may reflect a categorical distinction between face and scene processing. However, an alternative or complementary explanation is that they reflect differences in retinotopic eccentricity, which varies both within and between these regions. Spatial resolution varies systematically with eccentricity, with central vision supporting finer discriminations than the periphery. For example, face perception relies on high-acuity central vision, while scene perception draws on coarser information across the visual field. In the retina, cone-dense foveal regions supporting fine spatial vision show higher metabolic demands than rod-dominated peripheral regions. If this relationship carries forward to the visual cortex, including downstream IT cortex, it raises the possibility that CO intensity may covary with eccentricity representation within each region.

**Fig 2:**
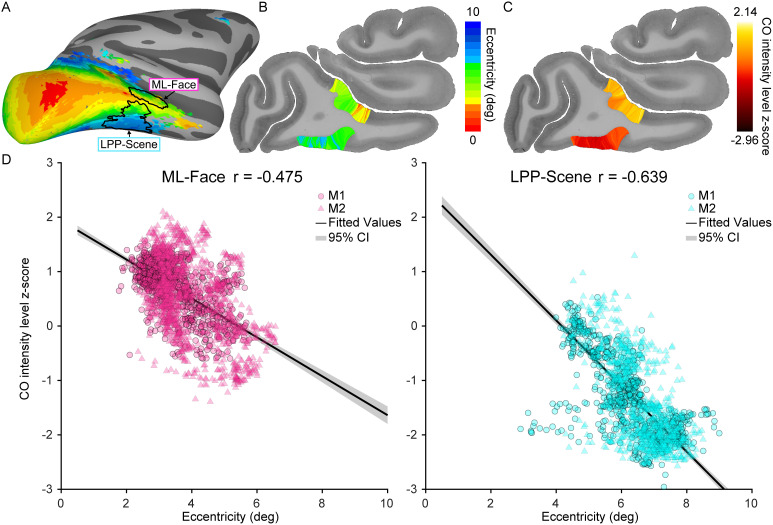
CO intensities are correlated with eccentricity in face and place patches. **(A)** A group average eccentricity map (*n* = 6) shown in the same surface view as Fig 1A. The color code indicates the preferred eccentricity at each cortical location, covering the central 10 degrees of visual space from center (red/yellow) to periphery (blue). The outlines of ML and LPP are overlaid on the cortical surface. **(B)** A representative histological section, illustrating the eccentricity representations in ML and LPP. Within ML and LPP, the cortex was divided into spatial bins oriented orthogonally to the cortical surface, and an eccentricity value was assigned to each bin based on the eccentricity map projected onto the histological data. **(C)** Mean CO intensities within the spatial bins in ML and LPP. CO intensities across cortical depth were averaged for each bin. **(D)** CO is correlated with eccentricity in ML (left) and LPP (right). Each point represents a spatial bin within the ROI (ML, magenta; LPP, cyan). Data from M1 and M2 were z-scored separately (M1: circles; M2: squares). Black lines show linear regression fits with gray shading indicating 95% confidence intervals. The data underlying this Figure can be found in https://osf.io/gjbmd.

To test this, we projected probabilistic maps of retinotopic eccentricity from fMRI onto the histological sections (Fig 2A). Within each ROI, the cortex was divided into spatial bins oriented orthogonally to the cortical surface. Although these bins do not correspond to anatomical cortical columns, they allowed localized sampling along the cortical ribbon. For each bin, we averaged CO intensity across cortical depth and extracted the corresponding eccentricity value (Fig 2B and 2C). This enabled direct comparison between local metabolic activity and retinotopic organization. Within both ML and LPP, CO intensity was significantly correlated with eccentricity (Pearson’s *r* = −0.475 and −0.639 for ML and LPP, respectively; both ps < 0.001; Fig 2D), with higher CO intensities in portions of cortex representing central visual space. This relationship was evident in both superficial and deep cortical portions within ML and LPP (all Pearson’s *r* ≤ −0.407; S1 Fig). These results indicate that differences in retinotopic organization contribute to the metabolic variation observed even within the category-selective cortex located downstream in the visual processing hierarchy.

### Eccentricity predicts CO intensity across visual cortex

**Fig 3:**
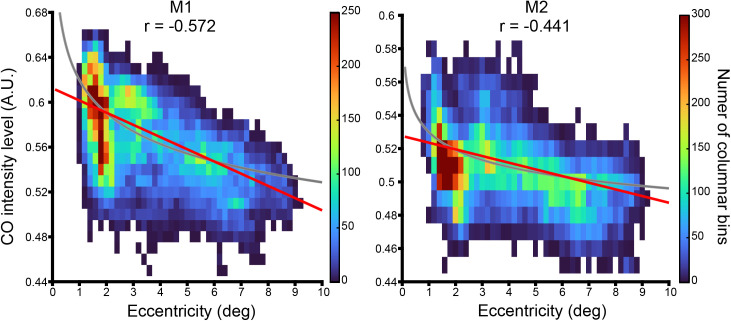
CO intensity varies systematically with eccentricity across the occipitotemporal visual cortex. Two-dimensional histograms show the distribution of spatial bins across occipitotemporal cortex as a function of eccentricity (bin width = 0.2 deg) and of CO intensity (bin width = 0.01) for monkey M1 (left) and M2 (right). Different CO intensity ranges and color scales were used for M1 and M2 to accommodate differences in CO signal range and the total number of columnar bins. Warmer colors indicate a higher number of spatial bins within each bin pair. Red lines indicate linear regression fits, and gray curves show low-order polynomial fits. In both monkeys, CO intensity is significantly correlated with eccentricity across the visual cortex. The data underlying this Figure can be found in https://osf.io/gjbmd.

Having established a local relationship between CO intensity and eccentricity within both face- and scene-selective regions, we next asked whether this relationship extends more broadly across the visual cortex. To address this, we expanded the spatial sampling analysis, dividing the full extent of the occipitotemporal cortex into bins oriented orthogonally to the cortical surface. For each bin, we derived average CO intensity and eccentricity values across cortical depth. This analysis revealed a significant correlation between CO and eccentricity in both monkeys (Pearson’s *r* = −0.572 for M1 and −0.441 for M2; both ps < 0.001; Fig 3), indicating that regions representing central visual space consistently exhibit higher metabolic activity. This relationship was evident in both superficial and deep cortical portions in both monkeys (all Pearson’s *r* ≤ −0.327, all ps < 0.001; S2 Fig), demonstrating that the observed relationship is not confined to a limited depth range. The correlations remained robust when using eccentricity maps from individual subjects instead of the probabilistic map (all Pearson’s r *≤* −0.369, all ps < 0.001; S3A Fig). These findings suggest that the relationship between CO intensity and eccentricity is a fundamental organizational feature across the visual hierarchy.

**Fig 4:**
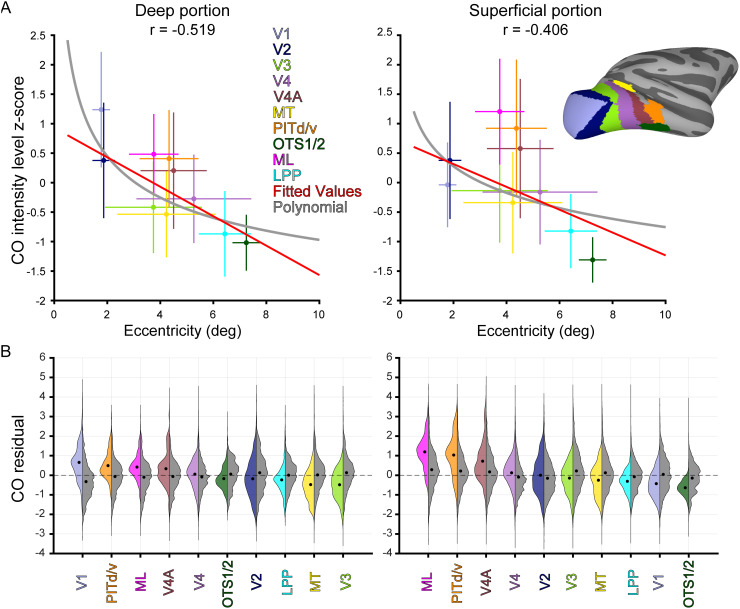
CO intensity variation across occipitotemporal areas reflects eccentricity biases, with additional effects in face-selective regions and input layers of V1. **(A)** Mean CO intensity and eccentricity for retinotopically defined visual areas are shown separately for deep (left) and superficial (right) portions of cortex. Each point represents the average value for one visual area, with error bars indicating standard deviation across spatial bins. Areas include V1, V2, V3, V4, V4A, MT, PITd/v, and OTS1/2. The colors of the points match the area labels shown in the left panel. The cortical surface in the right panel displays the anatomical locations of the visual areas. For reference, the points of ML (magenta) and LPP (cyan) are also shown; ML largely overlaps with PITd/v, and LPP with OTS1/2. Linear (red) and polynomial (gray) fits summarize the relationship between mean eccentricity and mean CO intensity across areas. **(B)** CO residual distributions for visual areas in deep (left) and superficial portions. Split violin plots comparing CO residual distributions for each visual area (left, colored by ROI) against eccentricity-matched null distributions (right, gray). Residuals represent deviations from the global robust linear regression of CO intensity on eccentricity. Null distributions were created by sampling voxels from all other visual areas with identical eccentricity profiles (5,000 iterations). Areas are ordered by mean residual magnitude (highest to lowest). Black circles indicate mean values for each distribution. The data underlying this Figure can be found in https://osf.io/gjbmd.

To examine how this CO-eccentricity relationship varies across the ventral visual hierarchy, we projected the probabilistic atlas of occipitotemporal retinotopic areas from V1 to PITd/v onto the histological sections. For each area, we then calculated the mean CO intensity and the mean eccentricity value to assess how these metrics change across the visual hierarchy. Across the ventral visual hierarchy, posterior-to-anterior decreases in CO were accompanied by shifts toward more peripheral eccentricity values, consistent with the known reduction in cortical magnification from V1 to higher visual areas [18,19]. These relationships were apparent for both deep and superficial portions of cortex (Pearson’s *r* = −0.519 for deep and −0.406 for superficial; both ps < 0.001; Fig 4A), indicating that the CO-eccentricity relationship spans the entire cortical depth. The correlations remained robust when using eccentricity maps from individual subjects instead of the probabilistic map (all Pearson’s *r* ≤ −0.323, all ps < 0.001; S3B Fig). Together, these findings reveal a systematic variation in metabolic activity across the visual hierarchy that is closely tied to retinotopic eccentricity.

Because spatial frequency (SF) and eccentricity are known to covary across the visual cortex [20,21], we asked whether SF is also associated with CO. Across all occipitotemporal cortex bins, SF and CO were significantly negatively correlated in both monkeys (M1, Pearson’s *r* = −0.463; M2, *r* = −0.347; both ps < 0.001; S4A Fig). However, these correlations were weaker than those observed between eccentricity and CO (Fig 3). In a multiple regression model with SF and eccentricity as predictors of CO, eccentricity accounted for more unique variance than SF, which accounted for relatively little unique variance (M1, partial *R*^2^ = 0.001 for SF and 0.143 for eccentricity, variance inflation factor (VIF) = 2.620; M2, partial *R*^2^ = 0.001 for SF and 0.087 for eccentricity, VIF = 2.908; S2A Table). Here, each partial R² was calculated by comparing model fit between the full model, which included both SF and eccentricity as predictors of CO, and reduced models in which one predictor was removed. Shared variance between SF and eccentricity was therefore not attributed uniquely to either predictor. Within category-selective regions, SF also showed weaker and less consistent relationships with CO than eccentricity did (ML, Pearson’s *r* = −0.342 [*p* < 0.001], partial *R*^2^ = 0.004 for SF and 0.130 for eccentricity, VIF = 2.610; LPP, *r* = 0.105 [*p* < 0.001], partial *R*^2^ = 0.027 for SF and 0.291 for eccentricity, VIF = 1.004; S4B Fig and S2B Table). Within LPP, the observed SF–CO correlation was small and opposite in sign to the negative correlations observed elsewhere, further suggesting that SF was not reliably associated with CO in this region. Together, these control analyses indicate that SF also covaries with CO, but more weakly than eccentricity, suggesting that eccentricity is more closely linked to the observed CO organization.

### Elevated CO in ML is not explained by central visual field bias

Although ML and LPP differ in category selectivity, they also differ in retinotopic bias, with ML representing more central and LPP more peripheral visual space. Because regions that represent central vision tend to show elevated CO intensity across the visual cortex, it is possible that the higher CO levels observed in ML simply reflect this central visual field bias. To test this, we used the linear model relating CO intensity to eccentricity described above and computed the standardized residuals for each columnar bin. We assessed whether individual ROIs deviated meaningfully from the global relationship between CO and eccentricity. We applied an eccentricity-matched null approach, which tests whether each area’s residual distribution differs meaningfully from what would be expected based on sampling from the broader visual cortex at matched eccentricities. For each ROI, we constructed a null distribution by sampling residual values from all other visual areas at matched eccentricity distributions, repeated 5,000 times to ensure stability (see Materials and methods).

This analysis revealed striking depth-specific patterns of metabolic organization (Figs 4B and S5 and S3 Table). In deeper cortical depth portions, V1 showed the most pronounced deviation from eccentricity expectations, with an adjusted effect size of 1.07 (95% CI: 1.035–1.105), explaining 23.3% of variance and showing only 54.5% distributional overlap with its matched null. This pattern is consistent with the high metabolic demands of layer IVc, which receives dense lateral geniculate nucleus (LGN) input. ML also showed elevated CO in deep portions (adjusted *d* = 0.893, 12.0% variance explained) but with greater distributional overlap (66.4%). In contrast, LPP showed a modest negative deviation in deep portions (adjusted *d* = −0.376) with high overlap (74.8%) and minimal variance explained (3.3%), indicating its residual distribution was largely indistinguishable from the eccentricity-matched null. In superficial portions, the pattern shifted dramatically. ML emerged as the strongest outlier (adjusted *d* = 1.10, 19.1% variance explained, 57.6% overlap), while V1 showed a negative deviation (adjusted *d* = −0.698, 8.0% variance explained). LPP again showed minimal meaningful deviation (adjusted *d* = −0.452, 3.4% variance explained, 79.3% overlap), suggesting its CO patterns largely conform to retinotopic expectations. These findings demonstrate that the elevated CO in ML cannot be attributed solely to its central visual field bias, as it shows pronounced deviations even when compared to other areas with matched eccentricity distributions.

### Category and eccentricity effects are not explained by tissue size

Although our analyses revealed strong relationships between CO intensity, category selectivity, and eccentricity, these effects could be influenced by systematic variability in staining across histological sections. Such variation can arise from technical artifacts during processing. Differences in tissue size across sections can lead to uneven staining, since smaller sections may absorb more stain when incubated for a fixed amount of time. Indeed, we observed a strong correlation between tissue size and CO intensity of the sections in both animals (Pearson’s *r* = −0.690 for M1 and −0.775 for M2). To control for this potential confound, we regressed CO intensity against tissue size across all columnar bins within the occipitotemporal cortex. We then used the residuals from this model, which reflect variation in CO orthogonal to tissue size, for all subsequent analyses. The differences in depth profiles between ML and LPP remained significant (all ts ≥ 2.697, all ps ≤ 0.011, FDR-corrected; S4 Table), as well as the correlations between CO intensity and eccentricity in ML and LPP (Pearson’s *r* = −0.672 and −0.617 for ML and LPP, respectively). The broader relationship between CO intensity and eccentricity across the visual cortex was also preserved (Pearson’s *r* = −0.342 for M1 and −0.303 for M2; *r* = −0.312 for superficial and −0.406 for deep). These findings confirm that the observed CO gradients and their functional associations are not artifacts of staining variability, but instead reflect intrinsic cortical organization.

## Discussion

Cortical specializations differ in both anatomical architecture and energetic demand. These differences are not randomly distributed, but reflect underlying organizational principles that structure the brain’s layout. In the visual system, one such principle is retinotopic eccentricity, which organizes cortical representations according to distance from the center of gaze and covaries with spatial resolution, receptive field properties, and category selectivity. Here, we show that cortical metabolic architecture, as indexed by CO intensity, also aligns systematically with eccentricity. Face- and scene-selective regions in IT cortex differed in CO intensity, and within each region, CO intensity covaries with eccentricity. More broadly, a continuous CO-eccentricity relationship spanned the occipitotemporal visual cortex. These results suggest that cortical metabolic resources are organized in relation to how visual information is sampled across space and the computational demands associated with that sampling.

### Mechanisms underlying eccentricity-dependent metabolic gradients

Our findings reveal a strong relationship between eccentricity and CO intensity. Why might cortical regions representing central vision be more metabolically demanding? One possibility is that neural circuits representing central visual space are specialized for higher-acuity processing and finer spatial discrimination than those representing peripheral space, and that these circuit properties carry greater sustained energetic demands. In line with this idea, retinal cones dominate the fovea and support high-acuity vision, but they do so at a steep energetic cost [22–24]. Their higher mitochondrial content, sustained ion exchange, and increased synaptic transmission result in elevated oxidative metabolism relative to peripheral rods [25,26]. Because retinotopic connectivity maintains distinct pathways for central and peripheral visual inputs through the visual hierarchy, differences in metabolic demand associated with these inputs could be reflected in cortical activity, thereby contributing to a foveal bias in cortical metabolic expenditure. This interpretation is consistent with classic demonstrations that CO expression reflects sustained, transsynaptic activity-dependent metabolic regulation [4,5].

The CO-eccentricity relationship may also be shaped by multiple co-aligned factors. Eccentricity covaries with several functionally relevant properties, including SF tuning, receptive field size, curvature sensitivity, and category selectivity [10,11,27,28]. Accordingly, the observed CO gradients should not be interpreted as uniquely attributable to eccentricity. Consistent with this, SF also covaried with CO, but these relationships were weaker and less consistent than those observed for eccentricity (S4 Fig). Another feature closely related to eccentricity, receptive field size, also does not provide a sufficient account of the observed metabolic gradients. Absolute receptive field size increases along the visual hierarchy from early visual cortex to IT [27,28], yet the face-selective region ML exhibited elevated CO levels relative to early visual areas, despite occupying a hierarchical position associated with larger receptive fields (Fig 4A). This dissociation further suggests that, although multiple co-aligned visual properties may contribute to the observed metabolic organization, eccentricity remains the more informative axis for interpreting the present CO pattern.

Neuronal metabolism is supported by coordinated vascular supply and molecular regulatory mechanisms. In V1, CO intensity correlates tightly with microvascular density, whereas its correlation with neuronal density is substantially weaker [29,30], suggesting that the observed regional variation in CO is not well explained by differences in cell packing density alone and instead more closely reflects differences in sustained energetic demand and its vascular support. Moreover, CO architecture has been shown to closely match the spatial distribution of NRF-2α, a transcription factor that regulates the expression of genes encoding CO, suggesting a tight coupling between metabolic architecture and transcriptional regulation [31,32]. Whether similar relationships between CO and both vascular and transcriptional specializations hold in category-selective regions of IT and across the visual cortex remains to be determined. Multimodal integration of CO histology with quantitative measures of these architectures across the visual hierarchy will be important for further testing the basis of the CO gradients observed here.

### CO as an index of metabolic architecture

The relationship between cortical metabolic architecture and functional organization has long been studied using a variety of metabolic mapping approaches in addition to CO histology. Classical histological studies using the 2-deoxyglucose (2-DG) method demonstrated spatially structured patterns of glucose utilization that closely correspond to functional architecture in visual cortex [33–36]. More recently, positron emission tomography (PET) using ^18^F-fluorodeoxyglucose (FDG) has enabled measurements of metabolic activity *in vivo* [34], and calibrated-fMRI approaches that estimate cerebral metabolic rate of oxygen (CMRO_2_) can non-invasively probe metabolic activity [37,38]. However, these methods primarily capture short-term metabolic responses associated with stimulus-evoked or ongoing neural activity. By contrast, CO histochemistry indexes sustained oxidative metabolic capacity and has been shown in the early sensory cortex to align with stable features of functional organization. In this context, our findings suggest that metabolic architecture also tracks large-scale functional organization across the visual hierarchy, making CO histochemistry particularly well-suited for studying stable cortical metabolic architecture.

### Region-specific CO architectures

Our depth-resolved residual analyses revealed region-specific depth signatures (Fig 4). V1 showed darker CO staining in deeper portions, consistent with the dense thalamic input to layer IVc [39]. In contrast, ML showed the darkest CO staining in more superficial portions. This pattern aligns with prior findings of pronounced CO staining in superficial portions of ML [2] and with anatomical evidence for dense corticocortical connectivity of this region with other face-processing regions [40]. Whether these depth-specific CO signatures reflect differences in input sources, local circuit properties, or processing demands remains an open question, but they highlight the potential of depth-resolved CO analysis to reveal how laminar and circuit-level organization contributes to functional specialization in higher-order visual cortex.

### Developmental and experiential influences

The observed eccentricity-dependent metabolic gradient raises the question of whether this gradient plays a formative role in cortical specialization or whether it simply reflects the demands of ongoing visual behavior. In macaques, adult-like CO patterns in V1 are present at birth [41] and have been linked to emerging functional distinctions [42]. While the organization of CO in the higher visual cortex at birth remains unstudied, retinotopic maps are well established before the appearance of category-selective regions in IT [21,43]. This developmental sequence suggests that early spatial and metabolic biases could constrain where functional specializations emerge. At the same time, visual experience likely contributes to metabolic organization. For example, infants spend a disproportionate amount of their early looking time fixating on faces, concentrating visual stimulation within central vision and potentially reinforcing eccentricity-related metabolic gradients [44]. This interpretation is supported by modeling work in which eccentricity-dependent energetic constraints gave rise to increased energy use in central regions, mirroring the cortical gradient observed in our data [45]. These perspectives are not mutually exclusive. Early metabolic biases may help guide the formation of specialized regions and remain embedded in the adult cortex, where they continue to support the demands of mature vision.

Studies of humans with altered early visual experience offer valuable opportunities to test how experience shapes metabolic organization. Individuals with reversed congenital cataracts show diminished foveal representation [46] and reduced face selectivity in inferior temporal cortex despite restored vision [47], suggesting long-lasting consequences of early visual deprivation for both retinotopic and category-selective organization. Examining metabolic architecture in such individuals could reveal whether the CO gradients we observe depend on typical early visual input. Together with experimental manipulations in animals, such as selective deprivation of specific visual features or training with novel stimuli [20], these approaches could clarify how visual experience shapes cortical metabolic organization. Integrating CO histology with developmental and experiential manipulations will help clarify how these metabolic gradients arise and are maintained.

### Broader implications for cortical organization

Recent gradient-based frameworks have provided valuable unifying perspectives on cortical organization across modalities, including functional connectivity, microstructure, receptor distributions, and gene expression [48,49]. Understanding how sensory topography relates to these broader organizational axes will be an important direction for future work, as direct links between them remain relatively underexplored and only limited studies have attempted to bridge sensory topography with large-scale intrinsic connectivity or molecular organization [50–53]. In this context, we interpret our findings as demonstrating that cortical metabolic architecture varies systematically along a well-established sensory topographic axis, without assuming a direct correspondence to whole-brain functional or molecular gradient frameworks.

Our primary comparison was between the face-selective region ML and the scene-selective region LPP, in order to test whether differences in metabolic architecture between ventral temporal regions could be understood in relation to retinotopic eccentricity. A broader implication of this framework is that the CO-eccentricity relationship may generalize beyond the ventral temporal cortex to other retinotopically organized visual regions, including the dorsal stream. Because dorsal and ventral regions differ systematically in magnocellular and parvocellular input biases, temporal dynamics, contrast sensitivity, and developmental trajectories [42,43,54,55], metabolic organization may also differ between streams. At the same time, ventral regions tend to be more foveally biased, whereas dorsal regions tend to be more peripherally biased [12,56,57]. Thus, any average metabolic differences between streams may reflect differences in retinotopic connectivity rather than stream identity itself. Consistent with this possibility, MT, a magnocellular-recipient dorsal-stream area, fell along the same CO–eccentricity relationship as ventral-stream regions (Fig 4A), suggesting that metabolic organization may vary with retinotopic eccentricity throughout visual cortex. Testing whether parietal regions with different eccentricity biases exhibit corresponding differences in CO intensity would provide a direct test of this hypothesis.

More broadly, our findings demonstrate the value of linking functional maps with metabolic architecture to uncover large-scale organizational principles of the brain. Neural computation is inherently metabolically expensive, yet few studies have directly examined how energy use is organized in relation to information-processing demands. By combining *in vivo* fMRI with *ex vivo* CO histology, we establish a direct link between behaviorally relevant cortical maps and their underlying metabolic architecture. Extending this approach to other sensory systems, as well as motor and cognitive domains, may reveal how structural and energetic constraints influence the development and organization of specialized brain functions. Ultimately, characterizing the energetic landscape of the brain may offer new insights into the biological cost of cognition and the pressures that shape its organization.

## Materials and methods

### Ethics statement

All procedures were approved in protocol (1,146) by the Harvard Medical School Institutional Animal Care and Use Committee (IACUC), following the Guide for the care and use of laboratory animals (Ed 8). This paper conforms to the ARRIVE Guidelines checklist.

### Non-human primates

All training, surgery, and experimental procedures were approved by the Harvard Medical School Animal Care and Use Committee and conformed with NIH guidelines for the humane care and use of laboratory animals. Two normally reared adult monkeys (Macaca mulatta) underwent T1w MRI and histological experiments. Nine normally reared adult monkeys underwent T1w and functional MRI.

### Histology and preprocessing

To quantify the neurometabolic architecture across the visual cortex, we analyzed the CO staining data from the two *ex vivo* macaque monkey brains, acquired in our previous study [2]. As previously reported [17], monkeys that had reached endpoints were euthanized by intravenous injection of SomnaSol (120 mg/kg sodium pentobarbital), and transcardially perfused with a rinse solution (0.9% sodium chloride and 0.5% sodium nitrite) followed by 4% paraformaldehyde in 0.1 M phosphate buffer (pH 7.4). After overnight postfixation, brains were transferred into 30% sucrose in 0.1 M phosphate buffer. Right hemisphere tissue was sectioned sagittally at 50-μm thickness using a freezing microtome, covering visual areas from V1 to IT. The left hemisphere was sectioned in a coronal orientation, only covering a small part of the IT anterior to V4, and was not included in this study. Serial sections were mounted on glass slides, postfixed in formol saline (10% formalin with 9 g/L sodium chloride) for 12 days, and processed for CO using standard protocols with catalase, cytochrome *c*, and 3,3′-diaminobenzidine reagents.

Digital images of CO-stained sections were captured using a Panasonic Lumix DMC-ZS7 camera with a 12× optical zoom under uniform lighting with a light box. Images were converted to 8-bit grayscale and normalized from 0–255 to 0–1 range, where 0 indicates no CO staining and 1 indicates the darkest staining. To correct for variability in the staining intensity across slices, we used white matter as a within-slice reference. For each section, we subtracted the difference between the section’s mean white matter intensity and each gray matter pixel’s intensity. To preserve the original intensity scale, we then added back the grand mean white matter intensity averaged across all sections.

Cortical CO architectures were quantified as a function of location on the cortical surface and depth by segmenting the gray matter into small spatial units using LayNii (https://github.com/layerfMRI/LAYNII; [58]). For each sagittal section, we used the LN_COLUMNAR_DIST tool to generate columnar bins oriented perpendicular to the cortical surface. Gray matter was further divided into 15 equivolume depth bins based on local cortical thickness. The number of depth bins was validated in our previous study [2], where 15 bins was the minimum required to consistently isolate the prominent CO band in primary visual cortex into 1–2 bins across sections, demonstrating that this resolution is sufficient to sample broad depth-dependent variation across superficial, intermediate, and deep portions of cortex, though the bins do not correspond to cytoarchitectonic laminae.

A 3D histological volume was reconstructed from serial sections as previously described [2]. In brief, brain tissue was segmented from the background using Illustrator (Adobe Systems) and converted to 8-bit grayscale images. To reduce computational demands while preserving resolution across cortical depth, images were downsampled to 768 × 1,024 pixels for sagittal sections and 1,024 × 768 pixels for coronal sections. Co-registration proceeded in two stages using MATLAB. First, a rigid-body alignment based on brain mask contours was applied across serial sections, using a middle slice as the reference. Second, gray matter was segmented from white matter using multilevel thresholding and used to refine alignment based on tissue contrast. These transformations were applied to the original CO-stained images, which were then stacked to form a 3D volume. To correct for inter-slice intensity fluctuations due to staining variability, we applied a section-wise intensity normalization procedure: we fit a quadratic curve to the mean section intensities and adjusted each slice to reduce deviations from the fitted trend, preserving gradual anatomical variation while minimizing technical artifacts.

### Creating and projecting (i) probabilistic face and place patches, (ii) eccentricity maps, (iii) visual areas from probabilistic atlases, and (IV) probabilistic spatial frequency maps

To define the face- and scene-selective regions in the IT cortex, as well as retinotopic organization, we used a probabilistic group atlas generated from independent fMRI datasets (*n* = 6 for face patches, *n* = 9 for place patches, *n* = 6 for retinotopy). Although the histology monkeys had individual face-patch localizations available from our prior work [2], they were not part of the independent fMRI datasets used to generate the probabilistic place-patch or retinotopy maps. Comparable individual localizations were therefore not available for place-patch or retinotopic ROIs. Atlas-based definitions were therefore used throughout so that face, place, and retinotopic comparisons were performed under the same analysis constraints across ROIs. In our prior study, probabilistic face-patch definitions preserved the key qualitative CO staining patterns observed with individually defined face patches, although with reduced effect sizes [2]. These atlases were originally constructed in the National Institute of Mental Health macaque template (NMT) brain space [11,17,59]. In the generation of these atlases, face- and scene-selective voxels were defined as clusters of >10 adjacent voxels that responded more strongly to images of faces compared to inanimate objects, or to images of scenes compared to images of other categories, respectively, as determined using a voxel-wise GLM (*p* < 0.0001, FDR-corrected). The probabilistic maps of the ML and LPP were created by calculating the percentage of subjects included in each voxel of NMT brain. For subsequent analyses, the probabilistic maps were thresholded at 70% to retain voxels with high inter-subject consistency. To align these maps with the histological data, we performed nonlinear registration between the NMT T1-weighted structural MRI and the reconstructed 3D histological volume using the JIP Analysis Toolkit (https://www.nitrc.org/projects/jip). Full details of this procedure are described in Oishi and colleagues (2024; [2]).

We used the same registration pipeline to project group-average probabilistic maps of retinotopic eccentricity and visual area boundaries, generated from six macaques ([11,60]; available at https://github.com/mikearcaro/BrainMaps/tree/main/Macaque/Retinotopy and https://brainana.readthedocs.io). To ensure that our eccentricity analyses focused on reliable and consistent responses, we applied spatial thresholds to include only voxels with a group-average F-statistic greater than 11 and a standard deviation in the eccentricity across macaques less than 3 degrees (Fig 2A). The histological sections included in this study span the occipitotemporal visual areas, including visual areas V1, V2, V3, V4, MT, PITd/v, OTS1/2. Face patch ML and place patch LPP largely overlap with PITd/v and OTS1/2, respectively [11,12].

We used the same registration pipeline to project group-average SF maps onto the histological sections for control analyses. These SF maps were generated from fMRI data in four macaques using a previously published phase-encoded SF-mapping protocol [21]. In that protocol, stimuli swept across eight logarithmically sampled SF values (3, 2, 1, 0.8, 0.5, 0.4, 0.2, and 0.1 cycles per degree [cpd]), and voxel-wise SF preferences were estimated using a Fourier-based analysis analogous to the retinotopy pipeline. SF preference maps were then averaged across subjects. To facilitate comparison with CO intensity, voxel-wise SF preferences were rescaled to a continuous range of 0–100 (where 0 corresponds to 3 cpd and 100 corresponds to 0.1 cpd) using a logarithmic transform that preserves the relative spacing of the sampled SF values. We restricted the analysis to voxels with a group-average *F*-statistic greater than 5. For the present study, we projected the group-average SF maps onto the reconstructed histological volume using the same nonlinear registration procedure used for the eccentricity and visual-area maps. For SF-CO correlation analyses, we used columnar bins overlapping the SF map. For multiple regression analyses, we restricted the sample to columnar bins overlapping both the SF and eccentricity maps. In the regression analyses, SF and eccentricity served as predictors of CO.

### CO profile comparisons between face and place patches

After projecting the face and place patches onto the histological sections, we quantified CO intensity within each ROI. Because nonlinear warping can distort ROI boundaries and lead to incomplete coverage across cortical depth, we redefined each ROI directly in the histological volume by selecting columnar bins that intersected the projected ROI on the cortical surface and extending each bin through all 15 depth levels. This approach ensured complete sampling of cortical depth within each ROI across all sections.

Within each histological section, we averaged CO intensities across columnar bins within each depth bin, yielding a depth-resolved CO profile for both ML and LPP. To quantify overall metabolic activity per ROI per section, we then averaged these profiles across all 15 depth bins, resulting in a single section-level intensity measure. These section-level intensity values were compared between ML and LPP patches using two-tailed unpaired t-tests (Fig 1D). To assess whether differences were specific to cortical depth, we repeated this comparison for superficial (mean of bins 1–7) and deep (mean of bins 8–15) depth segments. All comparisons were FDR-corrected for multiple testing.

### Correlations between CO and eccentricity in face and place patches

To examine the relationship between CO intensity and retinotopic eccentricity within ML and LPP, we used each columnar bin within a patch as an individual data point. For each bin, we calculated mean CO intensity by averaging across all 15 depth bins. The corresponding eccentricity value was derived from the projected probabilistic eccentricity map. Because the fMRI-derived eccentricity map reflects a surface-based projection while CO data span cortical depth, we assigned each columnar bin a single eccentricity value based on its surface location. To combine data across monkeys, we first z-scored the CO intensity and eccentricity values separately for each monkey (M1 and M2), then pooled the normalized data to calculate Pearson correlations between CO intensity and eccentricity across all columnar bins within each ROI. We repeated this analysis for superficial and deep portions by averaging CO intensity across the top 7 and bottom 8 depth bins, respectively, while retaining the corresponding surface-based eccentricity value for each columnar bin.

### CO–eccentricity relationships across visual cortex

To assess the relationship between CO intensity and eccentricity across the visual cortex, we computed the Pearson correlation coefficient across all columnar bins that overlapped with the group-average fMRI-defined eccentricity map. Consistent with the within-patch analysis, this analysis was performed across the full cortical depth and separately for superficial (bins 1–7) and deep (bins 8–15) segments for each monkey. We fit both linear and second-order polynomial regressions between CO intensity and eccentricity. To assess depth-specific effects, we repeated these analyses separately for the superficial (bins 1–7) and deep (bins 8–15) segments, averaging the z-scored results across monkeys.

To identify visual areas that significantly deviated from the cortex-wide CO-eccentricity relationship, we computed standardized residuals from a robust linear regression (bisquare weighting; MATLAB’s nlinfit) of CO on eccentricity across all columnar bins. For each area (including ML and LPP), we calculated the mean of these residuals across its constituent columnar bins, separately for superficial and deep segments. With thousands of columnar bins per area, even tiny deviations from the expected relationship can achieve statistical significance despite representing negligible biological effects. Moreover, areas naturally vary in their intrinsic CO variability, making it difficult to distinguish genuine functional specialization from sampling noise or differences in measurement precision. To distinguish statistically detectable differences from biologically meaningful ones, we developed an eccentricity-matched null approach for each ROI. We binned each ROI’s eccentricity distribution into 0.5° intervals and sampled the exact same number of residual values from each bin across all other visual areas, repeated 5,000 times to create stable null distributions. This preserved retinotopic structure while testing for area-specific metabolic patterns. We quantified practical significance using three complementary metrics:

1. within-ROI adjusted effect size standardized by the target ROI’s variance [*d*_adj = (*μ*_real − *μ*_null)/*σ*_real] with 95% bootstrap confidence intervals generated by resampling both distributions 1,000 times with replacement,
2. distribution overlap computed as the area under the minimum of kernel density estimates [∫min(*f*_real(*x*), *f*_null(*x*))*dx*],
3. proportion of variance explained using eta-squared [*η*^*2*^ = SS_between/SS_total] where SS_between represents variance between real and null groups and SS_total represents total variance in the combined sample.

All metrics used identical null subsamples (matched to real ROI size) to ensure internal consistency. Areas were considered meaningfully different when showing low distributional overlap (<0.7), large adjusted effect sizes (|*d*_adj| > 1.0, indicating deviations exceeding the ROI’s intrinsic variability), and substantial variance explained (>5%), addressing the challenge that large sample sizes make traditional significance tests overly sensitive to trivial differences.

### Correction for tissue size effect

To rule out confounds related to potential staining variability due to the difference in tissue size across sagittal sections, we modeled and regressed out the effect of tissue size on CO intensity. We regressed CO intensity against tissue size of the sections across all columnar bins in the occipitotemporal cortex for each monkey. The resulting residuals, representing variance in CO independent of tissue size effect, were used in control analyses to confirm that the observed CO-functional relationships could not be explained by systematic staining artifacts. In this control analysis, we used raw CO intensity values without the white matter normalization described above (where we subtracted the difference between each section’s mean white matter intensity and individual gray matter pixel intensities) to avoid potential overcorrection that might artificially introduce or mask correlations between tissue size and CO staining patterns.

## Supporting information

S1 Fig CO-eccentricity correlations in face- and scene-selective regions across cortical depth. Correlations are shown for ML (left) and LPP (right), with superficial portions shown in the top row and deep portions shown in the bottom row. Conventions are as in Fig 2D. The data underlying this Figure can be found in https://osf.io/gjbmd.
(TIF)

S2 Fig CO-eccentricity correlations across the occipitotemporal visual cortex are robust across cortical depth. Each panel represents a two-dimensional histogram with linear regression and low-order polynomial fits (red lines and gray curves, respectively). Pearson’s r is indicated in each panel. Conventions are as in Fig 3. Significant correlations are observed in both superficial (upper) and deep (lower) depth portions in M1 (left) and M2 (right). The data underlying this Figure can be found in https://osf.io/gjbmd.
(TIF)

S3 Fig CO–eccentricity correlations computed using eccentricity values from each individual fMRI subject (*N* = 6) were consistently negative. Filled circles denote correlations obtained using individual eccentricity maps (*N* = 6), whereas the open circle denotes the correlation obtained using the probabilistic eccentricity map. **(A)** Correlations across the occipitotemporal visual cortex in M1 (left) and M2 (right), related to Fig 3. **(B)** Correlations across the occipitotemporal visual cortex in deep (left) and superficial (right) depth portions, related to Fig 4A. The correlations consistently deviated from zero (all Pearson’s *r* ≤ −0.323, all ps < 0.001). This convergence supports the robustness of the CO–eccentricity relationship despite the lack of same-animal physiological mapping. The data underlying this Figure can be found in https://osf.io/gjbmd.
(TIF)

S4 Fig Spatial frequency (SF) covaries with CO, but more weakly and less consistently than eccentricity. **(A)** SF–CO correlations across occipitotemporal cortex. The inset image shows a group average SF map (*n* = 4) shown in the same surface view as Fig 2A. The color code indicates the preferred SF at each cortical location, covering a continuous range of 0–100 (where 0 corresponds to 3 cpd and 100 corresponds to 0.1 cpd). Two-dimensional histograms show the distribution of spatial bins across occipitotemporal cortex as a function of spatial frequency (bin width = 2 [A.U.], matched to the relative bin width used for eccentricity in Fig 3) and of CO intensity (bin width = 0.01) for monkey M1 (left) and M2 (right). Conventions are as in Fig 3. **(B)** SF-CO correlations within ML (left) and LPP (right). Conventions are as in Fig 2D. Pearson’s *r* is indicated in each panel (all ps < 0.001). The data underlying this Figure can be found in https://osf.io/gjbmd.
(TIF)

S5 Fig CO residual distributions for each visual area across cortical depth. Each panel displays the overlap between the CO residual distribution for each visual area (colored by ROI) and the eccentricity-matched null distribution (gray), with superficial portions shown in **(A)** and deep portions shown in **(B)**. Panels are ordered by the mean residual magnitude of the areas (highest to lowest). The distributions are identical to those shown in Fig 4B. The data underlying this Figure can be found in https://osf.io/gjbmd.
(TIF)

S1 Table Comparisons of CO profiles between face patch ML and place patch LPP, related to Fig 1D. Comparisons were performed using two-tailed unpaired *t*-tests collapsed across all 15 depth bins (whole depth), superficial (mean of bins 1–7) and deep (mean of bins 8–15) depth segments. FDR correction was applied to account for multiple comparisons. The data underlying this Table can be found in https://osf.io/gjbmd.
(XLSX)

S2 Table Multiple linear regression results with spatial frequency and eccentricity as predictors of CO. Multiple linear regressions were performed across all occipitotemporal cortex bins in both monkeys **(A)** and within face patch ML and place patch LPP **(B)**. SE, standard error; CI, confidence interval; RMSE, root mean squared error; VIF, variance inflation factor. Partial *R*^2^ indicates the unique variance explained by each variable after accounting for the other predictor. VIF was used to assess multicollinearity. The data underlying this Table can be found in https://osf.io/gjbmd.
(XLSX)

S3 Table Statistical difference between CO residual and eccentricity-matched null distributions for each visual area in Figs 4B and S5. Adjusted effect size represents the standardized d-prime and CI_Low and CI_High represent the 95% bootstrap confidence intervals. Overlap represents the proportion of the distribution overlap, computed as the area under the minimum of kernel density estimates. Variance explained represents the proportion of eta-squared. See Materials and methods for the details of the indices. Data are ordered by mean residual magnitude of the visual areas (highest to lowest). The data underlying this Table can be found in https://osf.io/gjbmd.
(XLSX)

S4 Table Comparisons of CO profiles between face patch ML and place patch LPP after correcting for the effect of tissue size on CO intensity. Conventions are as in S1 Table. The data underlying this Table can be found in https://osf.io/gjbmd.
(XLSX)

## Abbreviations

CO: cytochrome oxidase
FDG: ^18^F-fluorodeoxyglucose
FDR: false discovery rate
IT: inferotemporal
LGN: lateral geniculate nucleus
LPP: lateral place patch
ML: middle lateral
OXPHOS: oxidative phosphorylation
PET: positron emission tomography
ROIs: regions of interest
SF: spatial frequency
2-DG: 2-deoxyglucose
