# Supplementary figures and images for: Metabolic organization of macaque visual cortex reflects visual field topography and perceptual specialization
Source: PLoS Biol. 2026 Jun 8;24(6):e3003847. doi: 10.1371/journal.pbio.3003847 (PMC13286273; doi:10.1371/journal.pbio.3003847)

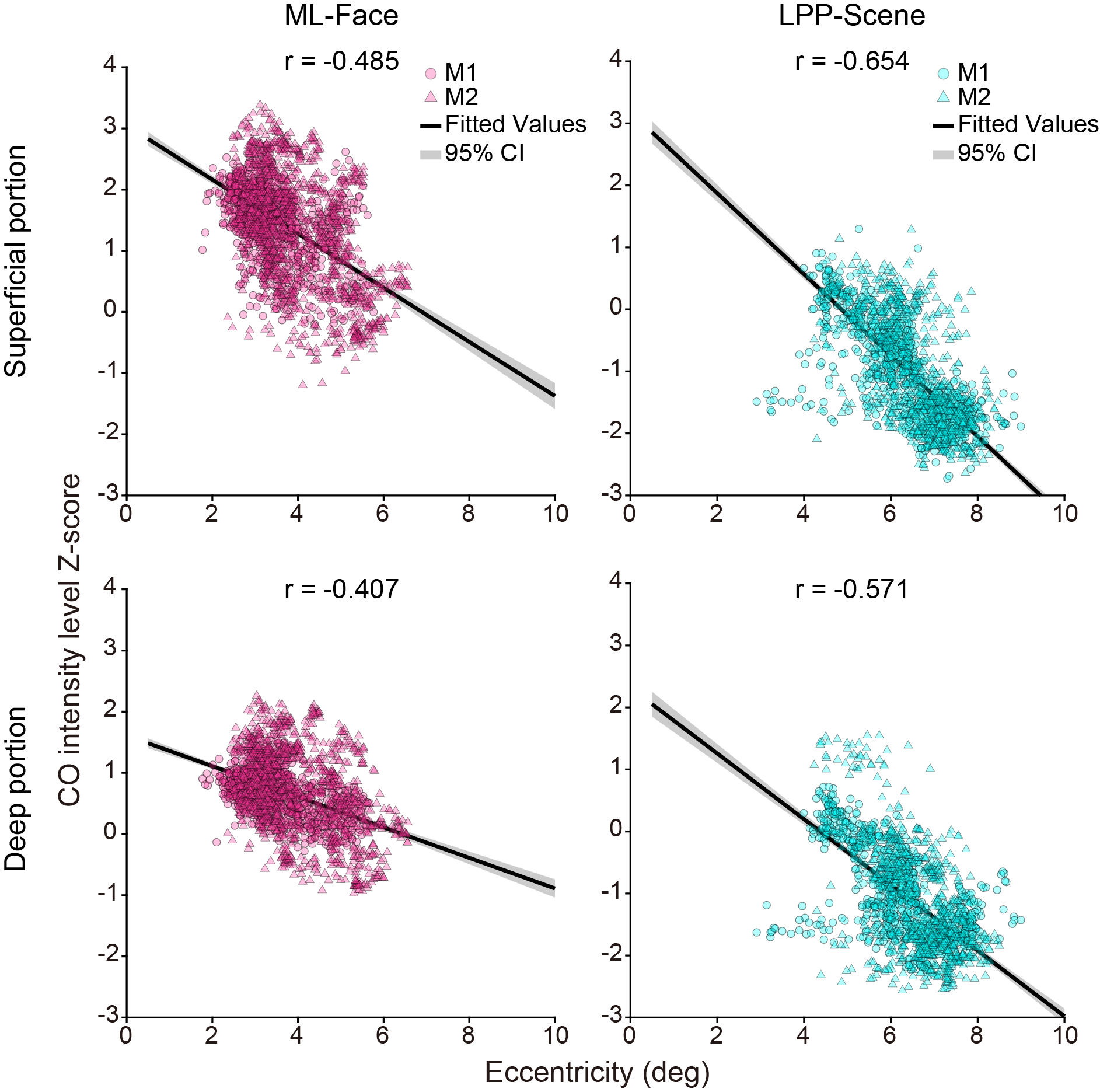

Supplement: S1 Fig — Correlations are shown for ML (left) and LPP (right), with superficial portions shown in the top row and deep portions shown in the bottom row. Conventions are as in Fig 2D. The data underlying this Figure can be found in https://osf.io/gjbmd. (TIF) [file pbio.3003847.s001.tif]

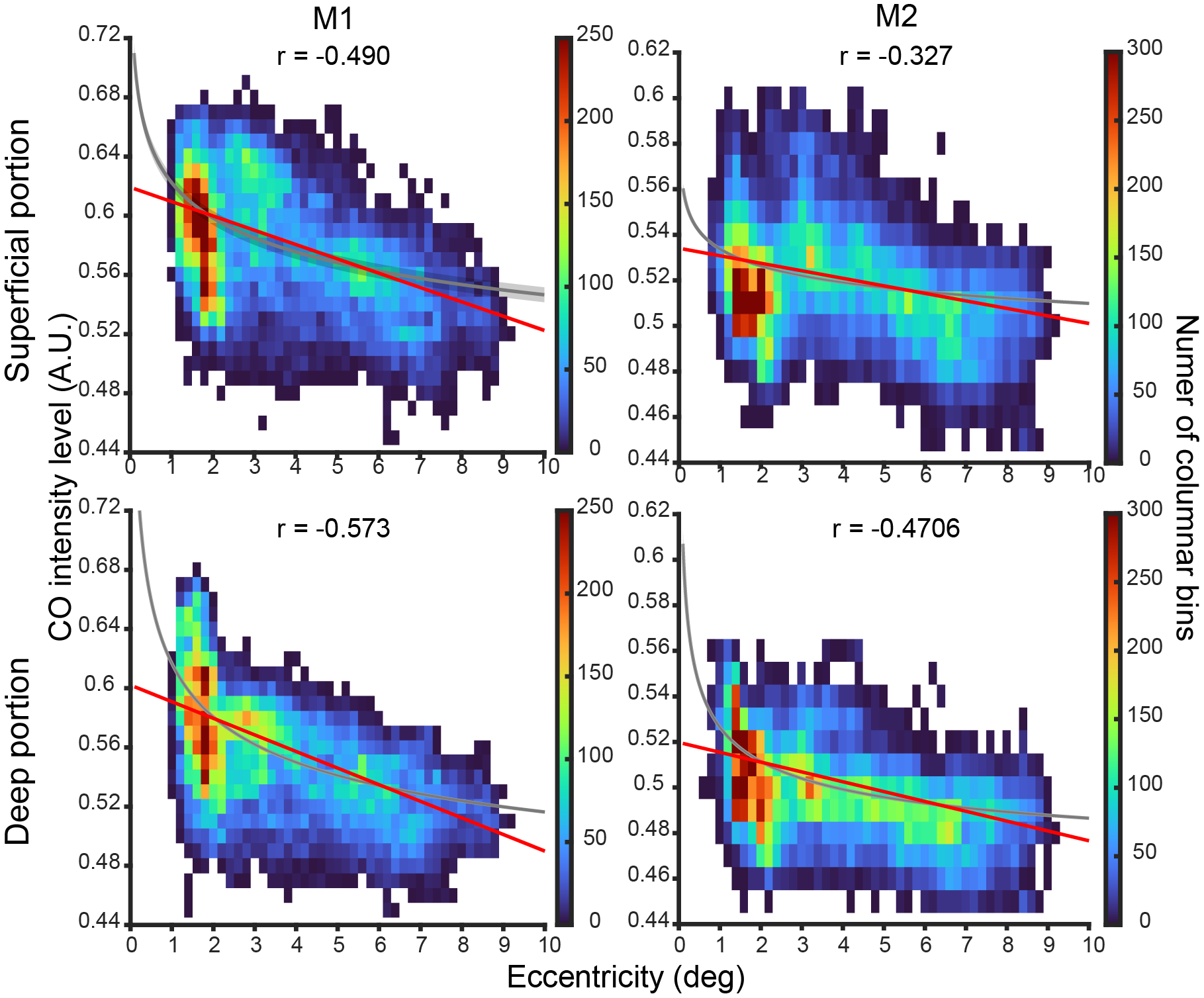

Supplement: S2 Fig — Each panel represents a two-dimensional histogram with linear regression and low-order polynomial fits (red lines and gray curves, respectively). Pearson’s r is indicated in each panel. Conventions are as in Fig 3. Significant correlations are observed in both superficial (upper) and deep (lower) depth portions in M1 (left) and M2 (right). The data underlying this Figure can be found in https://osf.io/gjbmd. (TIF) [file pbio.3003847.s002.tif]

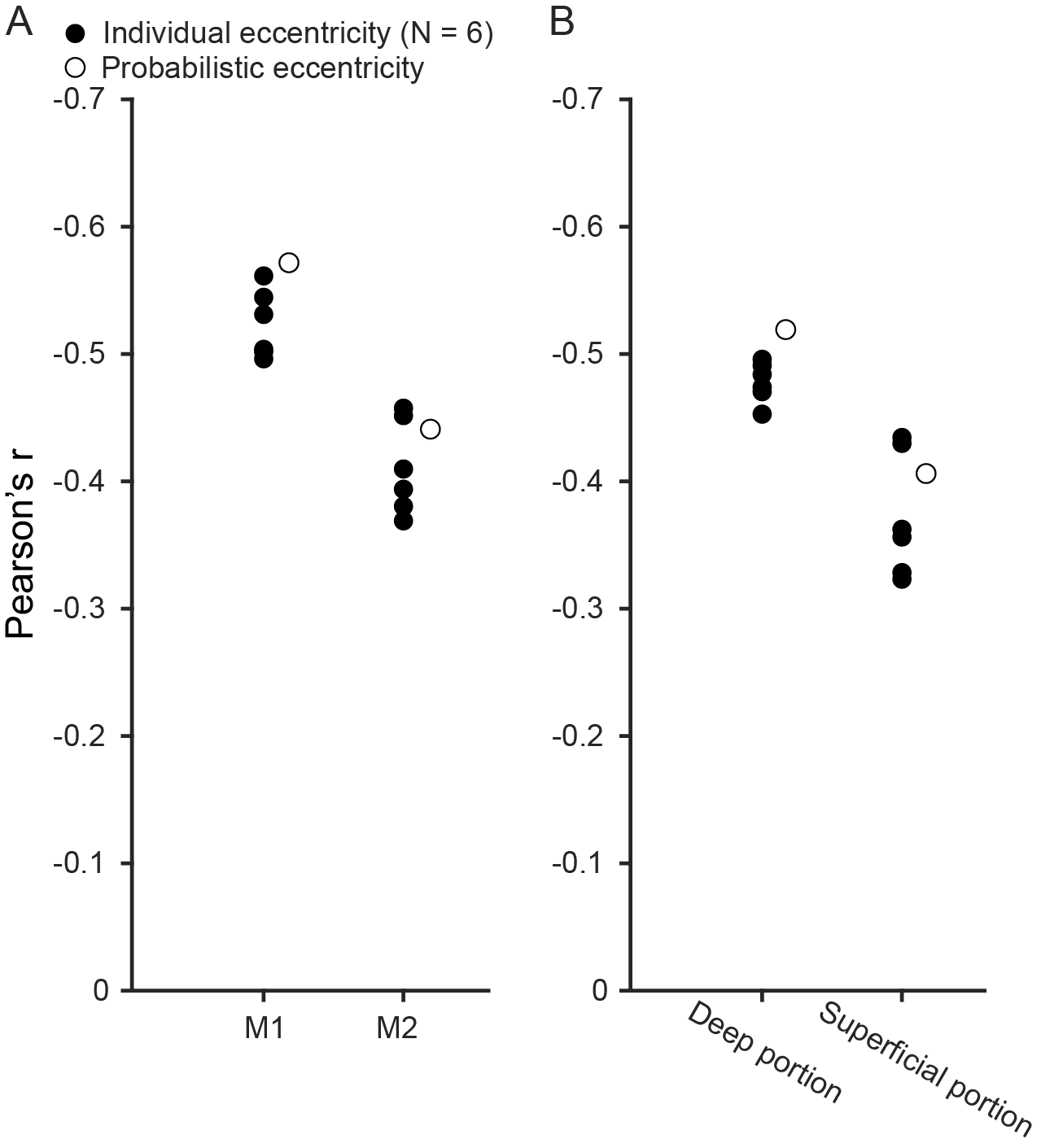

Supplement: S3 Fig — Filled circles denote correlations obtained using individual eccentricity maps (N = 6), whereas the open circle denotes the correlation obtained using the probabilistic eccentricity map. (A) Correlations across the occipitotemporal visual cortex in M1 (left) and M2 (right), related to Fig 3. (B) Correlations across the occipitotemporal visual cortex in deep (left) and superficial (right) depth portions, related to Fig 4A. The correlations consistently deviated from zero (all Pearson’s r ≤ −0.323, all ps < 0.001). This convergence supports the robustness of the CO–eccentricity relationship despite the lack of same-animal physiological mapping. The data underlying this Figure can be found in https://osf.io/gjbmd. (TIF) [file pbio.3003847.s003.tif]

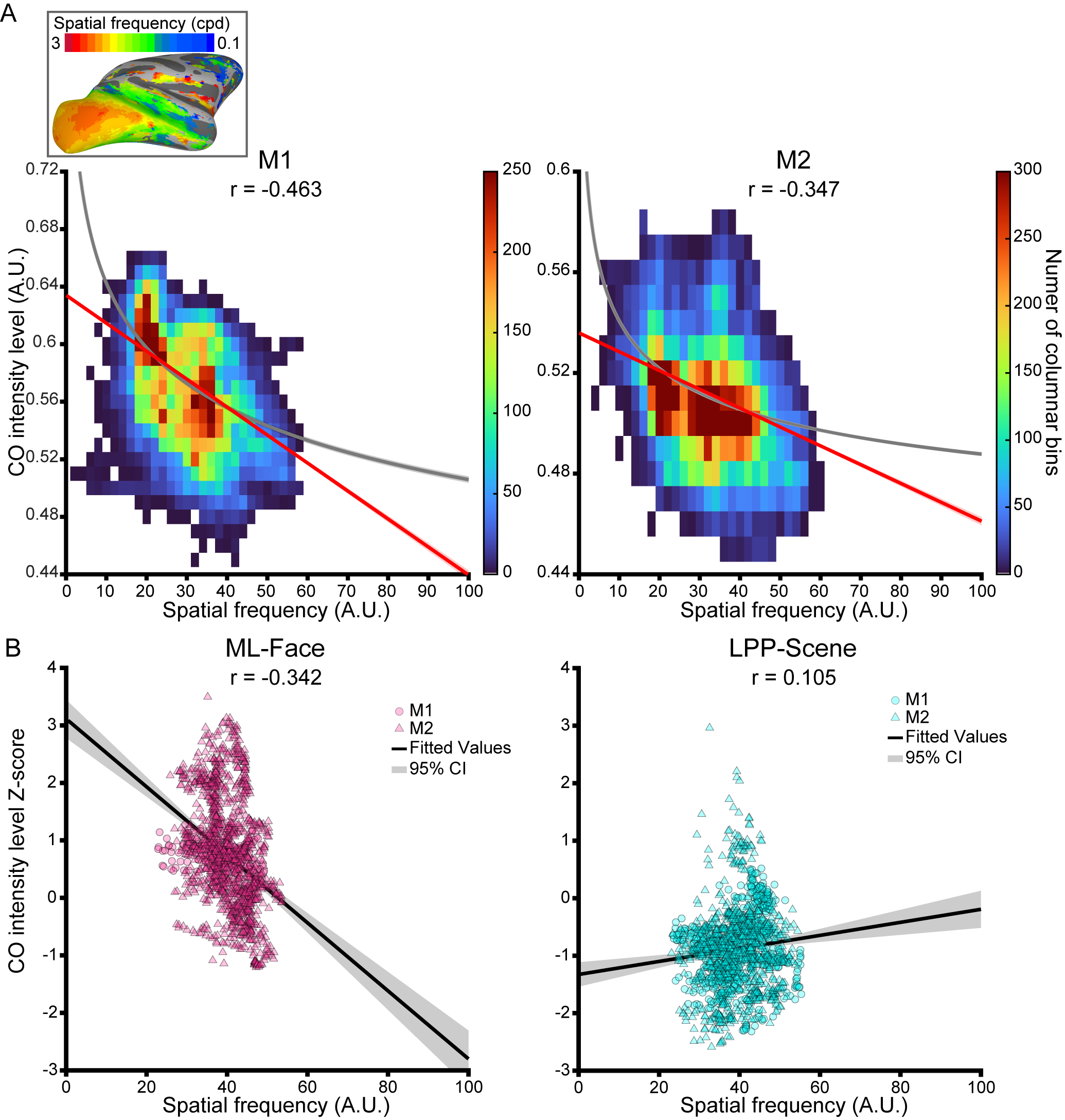

Supplement: S4 Fig — (A) SF–CO correlations across occipitotemporal cortex. The inset image shows a group average SF map (n = 4) shown in the same surface view as Fig 2A. The color code indicates the preferred SF at each cortical location, covering a continuous range of 0–100 (where 0 corresponds to 3 cpd and 100 corresponds to 0.1 cpd). Two-dimensional histograms show the distribution of spatial bins across occipitotemporal cortex as a function of spatial frequency (bin width = 2 [A.U.], matched to the relative bin width used for eccentricity in Fig 3) and of CO intensity (bin width = 0.01) for monkey M1 (left) and M2 (right). Conventions are as in Fig 3. (B) SF-CO correlations within ML (left) and LPP (right). Conventions are as in Fig 2D. Pearson’s r is indicated in each panel (all ps < 0.001). The data underlying this Figure can be found in https://osf.io/gjbmd. (TIF) [file pbio.3003847.s004.tif]

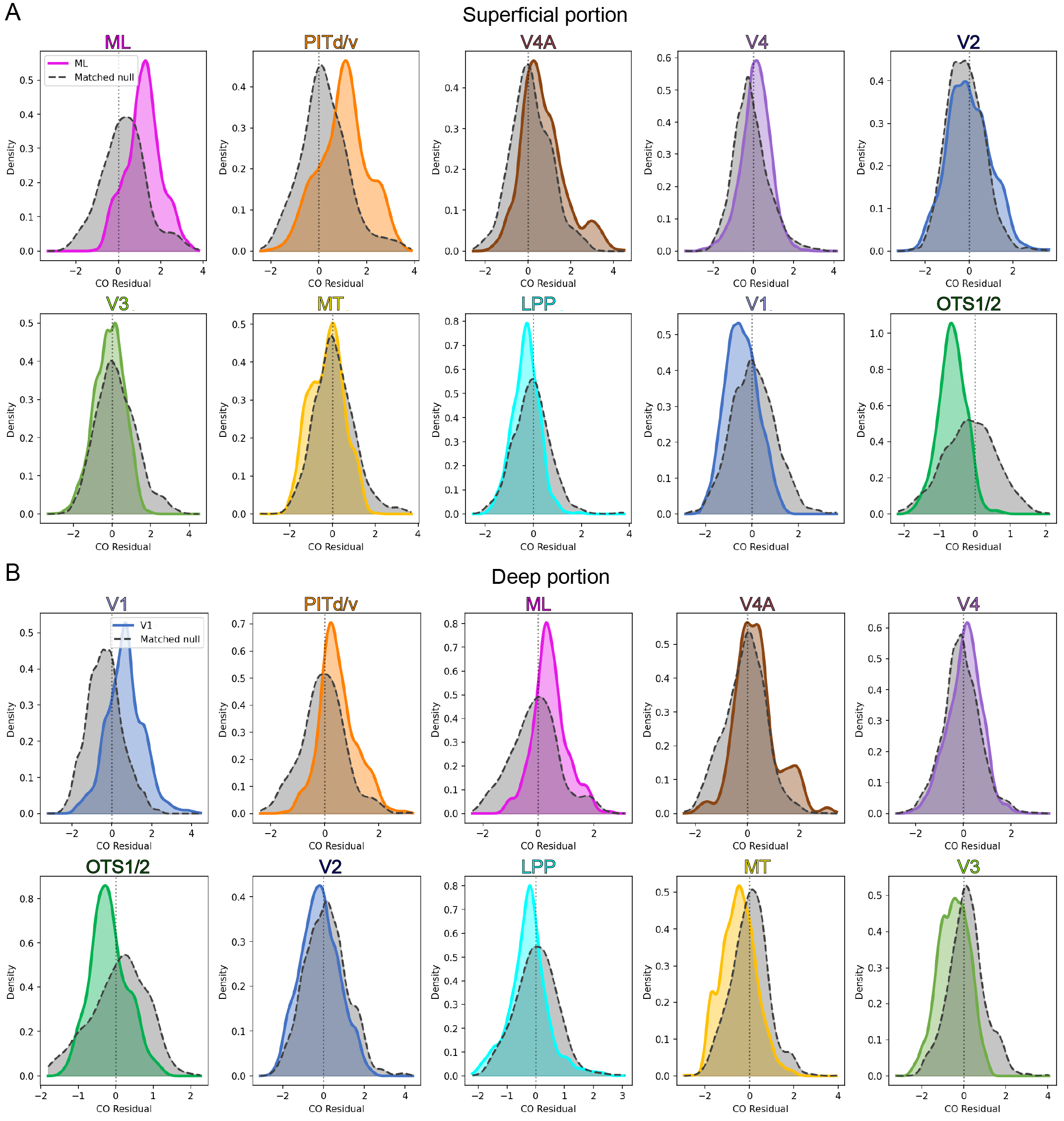

Supplement: S5 Fig — Each panel displays the overlap between the CO residual distribution for each visual area (colored by ROI) and the eccentricity-matched null distribution (gray), with superficial portions shown in (A) and deep portions shown in (B). Panels are ordered by the mean residual magnitude of the areas (highest to lowest). The distributions are identical to those shown in Fig 4B. The data underlying this Figure can be found in https://osf.io/gjbmd. (TIF) [file pbio.3003847.s005.tif]
