# Supplementary material for: Metabolic organization of macaque visual cortex reflects visual field topography and perceptual specialization
Source: PLoS Biol. 2026 Jun 8;24(6):e3003847. doi: 10.1371/journal.pbio.3003847 (PMC13286273; doi:10.1371/journal.pbio.3003847)
Supplement: Attachment — Submitted filename: Response to reviewers_PLOSBiology_Oishi_submission.docx [file pbio.3003847.s012.docx]

Dear Editors and Reviewers,

We would like to thank the editor and the three reviewers for their constructive comments, which helped us to improve the manuscript. Below, we address all comments point-by-point, discussing the subsequent revisions. Here, we have marked the comments in the decision letter in *blue*, and our text changes from the original submission in red. A reference list for all works cited in this Response is provided at the end of this document.

**Reviewers' Comments:**

Reviewer #1: A main point of the present manuscript is that face-selective region ML (biased toward central vision) exhibits higher cytochrome oxidase (CO) intensity than the scene-selective region LPP (biased toward peripheral vision). The Authors propose this difference is due to a close association of energy consumption with retinotopic eccentricity. They generalize as a second major point, that cortical metabolic resources are shaped by processing demands of visual perception. This is an interesting proposal, and the Authors are experienced and well positioned to address this technically and conceptually, by virtue of their several closely related previous publications. There are several points, however, for further clarification or improvement.

We thank the reviewer for this summary and the constructive criticism. Below, we address each comment, point by point.

1. The comparison of CO intensity and especially of depthwise differences are under documented; the textual description does not provide detail as to sublayers: "middle layers" are layer 4? or as in the literature, deep layer 3 and upper layer4 ? Or possible fluctuations along the tangential domain. Is there evidence of subdivisions within one or both areas? A figure would be helpful, similar to 2D in Arcaro et al., 2024 but at higher magnification.

We thank the reviewer for highlighting the need for clearer documentation of the depth-resolved CO analysis. We want to clarify at the outset that the 15 equivolume depth bins used in this study are not intended to identify cytoarchitectonic laminae or specific sublayers within ML or LPP. Accordingly, we do not make claims about layer-specific subdivisions or fine-grained tangential structure in these regions, and the present analysis is not designed to test for such subdivisions. Instead, our goal in using depth-resolved binning is to determine whether CO intensity differences are expressed uniformly across cortical depth or are preferentially weighted toward broad compartments, operationally defined as superficial versus deep portions of the cortex.

Two lines of evidence support the appropriateness of this approach for this purpose. First, in our prior study (Oishi et al., 2024, Cell Reports), we validated that 15 equivolume bins provide sufficient resolution to reliably isolate the prominent CO band in primary visual cortex (V1) into 1–2 bins across sections, demonstrating sensitivity to biologically meaningful depth-related variation without assigning cytoarchitectonic layer identity. Second, in the current dataset, depth-resolved analysis in V1 shows that the prominent CO band is darker than would be expected from the eccentricity-related CO gradient alone. This provides an internal validation that the approach captures meaningful depth-related variation without implying laminar specificity.

We agree that the original manuscript was insufficiently explicit on these points. We have revised the Methods to clarify that the depth bins do not correspond to cytoarchitectonic laminae, to avoid ambiguous terms such as “middle layers,” and to consistently refer instead to superficial and deep portions of cortex.

**Specific changes:**

In the Materials and Methods, we now state (p. 24, lines 499–504):

Gray matter was further divided into fifteen equivolume depth bins based on local cortical thickness. This number of depth bins was validated in our previous study [[2]](https://paperpile.com/c/rtlte2/W8cF), where 15 bins was the minimum required to consistently isolate the prominent CO band in primary visual cortex into 1–2 bins across sections, demonstrating that this resolution is sufficient to sample broad depth-dependent variation across superficial, intermediate, and deep portions of cortex, though the bins do not correspond to cytoarchitectonic laminae.

Why is the depth dimension even introduced? "These findings demonstrate that face- and scene-selective regions...differ in metabolic staining intensity, and that this difference spans the entire cortical depth."

We thank the reviewer for raising this important question. The depth-resolved analysis was introduced to determine whether regional differences in CO were distributed across the entire cortical column or driven by a narrower portion. Specifically, this analysis allowed us to determine whether the difference between face-selective ML and scene-selective LPP reflects a broad property across the entire cortical column or is driven by a narrow band of elevated CO. Empirically, we find that the difference is evident in both superficial and deep portions of cortex, motivating our statement that it “spans the entire cortical depth.”

To apply this depth-resolved approach consistently across all analyses, we further confirmed that the relationship between CO and eccentricity was evident in both superficial and deep cortical portions within face- and scene-selective patches (S1 Fig, related to Fig 2), as well as across the broader occipitotemporal visual cortex (S2 Fig, related to Fig 3).

Beyond this descriptive role, the depth-resolved analysis also proved biologically informative in the broader dataset. As we now discuss in the revised manuscript, region-specific depth signatures helped distinguish potentially different sources of metabolic demand: V1 showed darker CO in deeper portions, consistent with dense thalamic input to layer IVc, whereas ML showed relatively stronger superficial CO, consistent with dense corticocortical connectivity. Although the present depth bins do not imply laminar specificity, these findings illustrate that the depth dimension can reveal meaningful differences in cortical organization.

In the revised manuscript, we now explicitly state the motivation for the depth-resolved analysis and report additional results in the Results section and the methods in the Materials and Methods section.

**Specific changes:**

In the Results section (p. 6, lines 125–137):

To test whether category-selective regions differ in metabolic architecture, we compared the CO depth profiles between ML and LPP. In both regions, CO intensity increased from superficial to middle depths and then decreased toward deeper cortex, with peak staining in relatively superficial portions (Fig 1D). Although the overall depth profile was similar between regions, CO staining appeared consistently stronger in ML than in LPP across much of the cortical depth. We therefore asked whether this regional difference reflected a broad shift in CO intensity across depth or was restricted to a narrower depth range. To do so, we compared CO intensity between ML and LPP after averaging across the full cortical depth and separately within superficial and deep portions of cortex, defined as the top seven and bottom eight bins, respectively. ML exhibited significantly higher CO intensity than LPP in all three comparisons (all ts > 2.948, all ps < 0.001 except for the deep portion in M2, where p = 0.006, FDR-corrected; Fig 1D, S1 Table). These findings indicate that face- and scene-selective regions in IT differ in metabolic staining intensity, and that this difference is evident across both superficial and deep portions of cortex.

Also, in the Results section (p. 9, lines 175–178):

Within both ML and LPP, CO intensity was significantly correlated with eccentricity (Pearson’s r = -0.475 and -0.639 for ML and LPP, respectively; both ps < 0.001; Fig 2D), with higher CO intensities in portions of cortex representing central visual space. This relationship was evident in both superficial and deep cortical portions within ML and LPP (all Pearson’s r ≤ -0.407; S1 Fig).

Also, in the Results section (p. 10, lines 203–209):

This analysis revealed a significant correlation between CO and eccentricity in both monkeys (Pearson’s r = -0.572 for M1 and -0.441 for M2; both ps < 0.001; Fig 3), indicating that regions representing central visual space consistently exhibit higher metabolic activity. This relationship was evident in both superficial and deep cortical portions in both monkeys (all Pearson’s r ≤ -0.327, all ps < 0.001; S2 Fig), demonstrating that the observed relationship is not confined to a limited depth range.

In the Discussion section (p. 20, lines 393–401):

Our depth-resolved residual analyses revealed region-specific depth signatures (Fig 4). V1 showed darker CO staining in deeper portions, consistent with the dense thalamic input to layer IVc [[37]](https://paperpile.com/c/rtlte2/QQz6). In contrast, ML showed the darkest CO staining in more superficial portions. This pattern aligns with prior findings of pronounced CO staining in superficial portions of ML [[2]](https://paperpile.com/c/rtlte2/W8cF) and with anatomical evidence for dense corticocortical connectivity of this region with other face-processing regions [[38]](https://paperpile.com/c/rtlte2/xXit). Whether these depth-specific CO signatures reflect differences in input sources, local circuit properties, or processing demands remains an open question, but they highlight the potential of depth-resolved CO analysis to reveal how laminar and circuit-level organization contributes to functional specialization in higher-order visual cortex.

In Materials and Methods section (p. 28, lines 591–594):

We repeated this analysis for superficial and deep portions by averaging CO intensity across the top 7 and bottom 8 depth bins, respectively, while retaining the corresponding surface-based eccentricity value for each columnar bin.

Also, in Materials and Methods section (p. 28, lines 596–603):

To assess the relationship between CO intensity and eccentricity across the visual cortex, we computed the Pearson correlation coefficient across all columnar bins that overlapped with the group-average fMRI-defined eccentricity map. Consistent with the within-patch analysis, this analysis was performed across the full cortical depth and separately for superficial (bins 1–7) and deep (bins 8–15) segments for each monkey.

In Supplementary Information Legends (p. 38, lines 799–806):

**S1 Fig. CO-eccentricity correlations in face- and scene-selective regions across cortical depth.** Correlations are shown for ML (left) and LPP (right), with superficial portions shown in the top row and deep portions shown in the bottom row**.** Conventions are as in Fig 2D.

**S2 Fig. CO-eccentricity correlations across the occipitotemporal visual cortex are robust across cortical depth.** Each panel represents a two-dimensional histogram with linear-regression and low-order polynomial fits (red lines and gray curves, respectively). Pearson’s r is indicated in each panel. Conventions are as in Fig 3. Significant correlations are observed in both superficial (upper) and deep (lower) depth portions in M1 (left) and M2 (right).

2. Results Section 2 (CO intensity correlates with eccentricity.....). The proposed link of CO intensity to retinotopic organization is, as stated, rather speculative (despite last sentence in that section). One problem is the absence of physiological mapping in the same animal. The areas in these two animals are localized by comparison with previous studies and the CO pattern.

Another problem is the possible confound of multimodality. Finally, another problem becomes the heavy reliance on CO as a proxy for metabolic levels. There is a huge literature on category-selective patches (face or scene). Are there further corroborative data supportive of specifically metabolic differences; for example, cellular density? vascularity? receptor distribution? That would contribute fruther support, although perhaps something for the Discussion.

We thank the reviewer for raising these important concerns regarding interpretation, localization, and biological specificity. We agree that the relationship between CO intensity and retinotopic eccentricity must be framed carefully. Accordingly, we treat eccentricity throughout as a descriptive feature that helps constrain interpretation of metabolic variation, rather than as a unique or causal explanation for regional CO differences. Below, we address each of the reviewer’s subpoints regarding mapping, multimodality, and the interpretation of CO as a metabolic proxy.

**Absence of same-animal physiological mapping.**

We agree that obtaining retinotopic maps and CO histology from the same animals would provide the most direct test of the proposed relationship. At the same time, the anatomical localization of retinotopic eccentricity organization and of face- and scene-selective regions in macaque inferotemporal cortex is highly consistent across individual monkeys (Nasr et al., 2011 J Neurosci.; Kornblith et al., 2013 Neuron; Janssens et al., 2014 J Neurosci.; Arcaro and Livingstone, 2017 J Neurosci.; Arcaro et al., 2020 PNAS). At the spatial scale examined here, group-average eccentricity maps therefore provide a suitable reference for region-level analyses. The probabilistic face- and scene-selective regions used in this study were projected onto the histological sections via registration of the NMT template to each individual histological volume, rather than being defined directly within the histology animals themselves.

Support for this approach comes from our prior work directly combining *in vivo* fMRI with postmortem CO histology in the same animals. In that study, atlas-based region definitions recovered the same qualitative CO patterns as individual-subject definitions, albeit with reduced effect sizes (Oishi et al., 2024 Cell Reports). This convergence indicates that while same-animal physiological mapping improves precision, region-level variations of CO architecture can still be recovered using anatomically aligned reference maps.

To further address the concern regarding cross-animal alignment, we computed CO-eccentricity correlations using eccentricity maps from each individual fMRI subject separately (N=6) instead of using the probabilistic eccentricity map (S3 Fig below). Across all analyses corresponding to Figs 3 and 4, the correlations remained negative (all Pearson’s r ≤ −0.323). Effect sizes were generally reduced relative to those obtained using the probabilistic map, which is expected because individual-monkey maps are noisier estimates than the group average and do not benefit from signal averaging across monkeys. Together, these analyses strengthen confidence that the observed CO-eccentricity relationship is robust to cross-animal variation in the functional maps, while not removing the important limitation that retinotopic mapping and histology were not obtained in the same animals.

**Specific changes:**

In the Results, we write (p. 6, lines 116–118):

Functional regions of interest (ROIs) were defined using probabilistic maps of category selectivity from fMRI, with the locations of ML and LPP corresponding to those reported across laboratories [[11–15]](https://paperpile.com/c/rtlte2/LOBl+T1g5+eiWW+qK96+5Zf0u).

In the Results, we write (pp. 10–11, lines 203–211 and lines 219–223):

This analysis revealed a significant correlation between CO and eccentricity in both monkeys … The correlations remained robust when using eccentricity maps from individual subjects instead of the probabilistic map (all Pearson’s r ≤ −0.369, all ps < 0.001; S3 FigA).

These relationships were apparent for both deep and superficial portions of cortex (Pearson’s r = -0.519 for deep and -0.406 for superficial; both ps < 0.001; Fig 4A), indicating that the CO-eccentricity relationship spans the entire cortical depth. The correlations remained robust when using eccentricity maps from individual subjects instead of the probabilistic map (all Pearson’s r ≤ −0.323, all ps < 0.001; S3 FigB).

In the Supplementary Information Legends (p. 29, lines 807–815):

**S3 Fig. CO–eccentricity correlations computed using eccentricity values from each individual fMRI subject (N = 6) were consistently negative.** Filled circles denote correlations obtained using individual eccentricity maps (N = 6), whereas the open circle denotes the correlation obtained using the probabilistic eccentricity map. **(A)** Correlations across the occipitotemporal visual cortex in M1 (left) and M2 (right), related to Fig 3. **(B)** Correlations across the occipitotemporal visual cortex in deep (left) and superficial (right) depth portions, related to Fig 4A. The correlations consistently deviated from zero (all Pearson’s r ≤ −0.323, all ps < 0.001). This convergence supports the robustness of the CO-eccentricity relationship despite the lack of same-animal physiological mapping.

**Multimodality and co-aligned organizational factors.**

We agree that the ventral temporal cortex is organized along multiple correlated dimensions, including retinotopy, category selectivity, low-level visual tuning properties and hierarchical position, such that eccentricity does not operate in isolation (see Figure 1 in Arcaro & Livingstone, 2024 Annual Review of Neuroscience; Figure 4 in Grill-Spector & Weiner, 2014 Nature Reviews Neuroscience). Our goal is therefore not to attribute CO differences uniquely to eccentricity, but rather to demonstrate that metabolic architecture aligns systematically with a well-established retinotopic gradient. This is particularly relevant because retinotopy is present early in development (Arcaro et al., 2017, eLife), precedes the emergence of several other correlated functional dimensions (Arcaro et al., 2017 eLife, Arcaro et al., 2017 Nature Neuroscience and Livingstone et al. 2017 Nature Communications), and has been proposed to scaffold the later emergence of other functional properties (Arcaro and Livingstone, 2021 Nature Review Neurosci.). Even in a multimodal system, the finding that CO intensity covaries with eccentricity remains informative. It indicates that regions biased toward different portions of visual space differ in their long-term metabolic demands, thereby placing meaningful constraints on possible mechanistic accounts.

To evaluate whether the observed CO pattern might be related to a visual feature dimension that covaries with eccentricity, we analyzed a group-average spatial frequency (SF) map. Because SF covaries with eccentricity across the visual cortex (Srihasam et al. 2014, Nature Neuroscience) and is present early postnatally (Arcaro et al. 2017, eLife), it provides a useful comparison for testing whether the observed CO pattern reflects eccentricity or another closely related visual dimension. We used the same registration pipeline to project group-average SF maps onto the histological sections. These SF maps were generated from fMRI data in four macaques using a previously published phase-encoded SF-mapping protocol (Arcaro and Livingstone, 2017 eLife). Across all occipitotemporal cortex bins, SF and CO were significantly negatively correlated in both monkeys (M1, Pearson’s r = -0.463; M2, r = -0.347; S4 FigA). However, these correlations were weaker than those observed between eccentricity and CO (M1, r = -0.572; M2, r = -0.441; Fig 3). In a multiple regression model with SF and eccentricity as predictors of CO, eccentricity accounted for more unique variance than SF, which accounted for relatively little unique variance (M1, partial R^2^ = 0.001 for SF and 0.143 for eccentricity, VIF = 2.620; M2, partial R^2^ = 0.001 for SF and 0.087 for eccentricity, VIF = 2.908; S2 TableA). Within category-selective regions, SF also showed weaker and less consistent relationships with CO than eccentricity did (face-selective region ML, Pearson’s r = -0.342, partial R^2^ = 0.004 for SF and 0.130 for eccentricity, VIF = 2.610; scene-selective region LPP, r = 0.105, partial R^2^ = 0.027 for SF and 0.281 for eccentricity, VIF = 1.004; all ps < 0.001; S4 FigB, S2 TableB). Together, these analyses indicate that SF also covaries with CO, but more weakly than eccentricity.

Another feature closely related to eccentricity, receptive field size, also does not provide a sufficient account of the observed metabolic gradients. Absolute receptive field size increases along the visual hierarchy from early visual cortex to IT (Kravitz et al., 2013 TiCS; Kay et al., 2015 Current Biology), yet the face-selective region ML exhibited elevated CO levels relative to early visual areas despite occupying a hierarchical position associated with larger receptive fields (Fig 4A). This dissociation further suggests that, although multiple co-aligned visual properties may contribute to the observed metabolic organization, eccentricity remains the more informative axis.

To reduce the possibility of overinterpretation, we have expanded the Discussion to situate the CO–eccentricity relationship within a broader, multimodal framework of visual cortex organization and to clarify that eccentricity is not intended as a unique explanatory factor. At the same time, the additional SF analyses and the broader literature support the view that eccentricity remains a key organizing principle of metabolic variation.

**Specific changes:**

In the Results, we added the results of the correlation between SF and CO (pp. 12–13, lines 237–252):

Because spatial frequency (SF) and eccentricity are known to covary across the visual cortex [[18,19]](https://paperpile.com/c/rtlte2/0Xa2+ixIY), we asked whether SF is also associated with CO. Across all occipitotemporal cortex bins, SF and CO were significantly negatively correlated in both monkeys (M1, Pearson’s r = -0.463; M2, r = -0.347; both ps < 0.001; S4 FigA). However, these correlations were weaker than those observed between eccentricity and CO (Fig 3). In a multiple regression model with SF and eccentricity as predictors of CO, eccentricity accounted for more unique variance than SF, which accounted for relatively little unique variance (M1, partial R^2^ = 0.001 for SF and 0.143 for eccentricity, VIF = 2.620; M2, partial R^2^ = 0.001 for SF and 0.087 for eccentricity, VIF = 2.908; S2 TableA). Within category-selective regions, SF also showed weaker and less consistent relationships with CO than eccentricity did (ML, Pearson’s r = -0.342 [p < 0.001], partial R^2^ = 0.004 for SF and 0.130 for eccentricity, VIF = 2.610; LPP, r = 0.105 [p < 0.001], partial R^2^ = 0.027 for SF and 0.291 for eccentricity, VIF = 1.004; S4 FigB, S2 TableB). Within LPP, the observed SF-CO correlation was small and opposite in sign to the negative correlations observed elsewhere, further suggesting that SF was not reliably associated with CO in this region. Together, these control analyses indicate that SF also covaries with CO, but more weakly than eccentricity, suggesting that eccentricity is more closely linked to the observed CO organization.

In the Discussion, we write (p. 18, lines 350–362):

The CO-eccentricity relationship may also be shaped by multiple co-aligned factors. Eccentricity covaries with several functionally relevant properties, including SF tuning, receptive field size, curvature sensitivity, and category selectivity [23–26]. Accordingly, the observed CO gradients should not be interpreted as uniquely attributable to eccentricity. Consistent with this, SF also covaried with CO, but these relationships were weaker and less consistent than those observed for eccentricity (S4 Fig). Another feature closely related to eccentricity, receptive field size, also does not provide a sufficient account of the observed metabolic gradients. Absolute receptive field size increases along the visual hierarchy from early visual cortex to IT [[25,26]](https://paperpile.com/c/rtlte2/nsfH+O0HG), yet the face-selective region ML exhibited elevated CO levels relative to early visual areas, despite occupying a hierarchical position associated with larger receptive fields (Fig 4A). This dissociation further suggests that, although multiple co-aligned visual properties may contribute to the observed metabolic organization, eccentricity remains the more informative axis for interpreting the present CO pattern.

In the Materials and Methods, we write about the analysis using the probabilistic SF maps (pp 26–27, lines 552–566):

We used the same registration pipeline to project group-average spatial frequency (SF) maps onto the histological sections for control analyses. These SF maps were generated from fMRI data in four macaques using a previously published phase-encoded SF-mapping protocol [[19]](https://paperpile.com/c/rtlte2/ixIY). In that protocol, stimuli swept across eight logarithmically sampled SF values (3, 2, 1, 0.8, 0.5, 0.4, 0.2, and 0.1 cycles per degree [cpd]), and voxel-wise SF preferences were estimated using a Fourier-based analysis analogous to the retinotopy pipeline. SF preference maps were then averaged across subjects. To facilitate comparison with CO intensity, voxel-wise SF preferences were rescaled to a continuous range of 0–100 (where 0 corresponds to 3 cpd and 100 corresponds to 0.1 cpd) using a logarithmic transform that preserves the relative spacing of the sampled SF values. We restricted the analysis to voxels with a group-average F-statistic greater than 5. For the present study, we projected the group-average SF maps onto the reconstructed histological volume using the same nonlinear registration procedure used for the eccentricity and visual-area maps. Columnar bins that overlapped with the eccentricity maps were used for correlation analyses and for multiple regression analyses. In the regression analyses, SF and eccentricity served as predictors of CO.

In the Supplementary Information Legends (p. 38, lines 807–815 and lines 835–840):

**S4 Fig. Spatial frequency (SF) covaries with CO, but more weakly and less consistently than eccentricity. (A)** SF-CO correlations across occipitotemporal cortex. The inset image shows a group average SF map (n = 4) shown in the same surface view as Fig 2A. The color code indicates the preferred SF at each cortical location, covering a continuous range of 0–100 (where 0 corresponds to 3 cpd and 100 corresponds to 0.1 cpd). Two-dimensional histograms show the distribution of spatial bins across occipitotemporal cortex as a function of spatial frequency (bin width = 2 [A.U.], matched to the relative bin width used for eccentricity in Fig 3) and of CO intensity (bin width = 0.01) for monkey M1 (left) and M2 (right). Conventions are as in Fig 3. **(B)** SF-CO correlations within ML (left) and LPP (right). Conventions are as in Fig 2D. Pearson’s r is indicated in each panel (all ps < 0.001).

**S2 Table. Multiple linear regression results with spatial frequency and eccentricity as predictors of CO.** Multiple linear regressions were performed across all occipitotemporal cortex bins in both monkeys (A) and within face patch ML and place patch LPP (B). SE, standard error; CI, confidence interval; RMSE, root mean squared error; VIF, variance inflation factor. Partial R^2^ indicates the unique variance explained by each variable after accounting for the other predictor. VIF was used to assess multicollinearity.

**CO as a proxy for metabolic demand.**

We agree that CO has not been widely used to characterize metabolic organization beyond early sensory cortices, particularly in the inferotemporal cortex. However, CO is a well-established histochemical marker of sustained oxidative metabolic capacity, and in the macaque visual system, its distribution has been shown to correspond closely with microvascular density across cortical layers (Weber et al., 2008 Cerebral Cortex). This supports the interpretation of CO as indexing stable energetic demand rather than transient neural activity. We therefore view CO as a biologically grounded proxy for long-term metabolic architecture, while acknowledging that its application to category-selective regions in the inferotemporal cortex remains less developed than in earlier visual areas. We also agree that additional corroborative evidence would strengthen interpretation of metabolic differences across category-selective regions. However, histoarchitectonic and molecular features of these regions remain comparatively understudied, in large part because few studies have co-registered *in vivo* functional localization with postmortem histology (Grimaldi et al., 2016 Neuron; Oishi et al., 2024 Cell Reports).

In the revised manuscript, we clarify in the Introduction that CO is a validated marker of stable metabolic architecture, and expand the Discussion to situate this interpretation within converging vascular and transcriptomic evidence from the visual cortex. The specific manuscript changes addressing this point are described in full in our response to the related Comment 3 below.

3. As stated in the Discussion, the proposed relationship between eccentricity and CO intensity "may be shaped by multiple co-aligned factors." Instead of the rather general discussion that follows, a stronger argument might be to review evidence about CO and metabolism, including corroborative evidence from vascularity or (as mentioned above, receptor distribution? gene expression?) This is insufficiently reviewed in the present manuscript, even as relates to the basics of CO pre- or postsynaptic influence.

We thank the reviewer for this suggestion and agree that the Discussion should more directly link the biological basis of CO staining to the interpretation of our findings, particularly given our acknowledgment that the relationship between eccentricity and CO may be shaped by multiple co-aligned factors. We have revised the manuscript accordingly to clarify what CO does and does not reflect and how this constrains the conclusions of the present study.

In the context of our study, CO histochemistry reflects the long-term metabolic demand of synaptic processing. Foundational work by Wong-Riley and colleagues demonstrated that CO staining changes transsynaptically following sustained alterations in functional input and is preferentially associated with dendritic and postsynaptic metabolic demands (Wong-Riley and Welt, 1980 PNAS; Wong-Riley, 1989 TiCS), establishing that regional CO differences reflect stable metabolic architecture rather than short-lived stimulus-evoked activity.

Independent evidence further constrains this interpretation. In macaque primary visual cortex, CO activity correlates tightly with microvascular density across cortical layers, whereas its correlation with neuronal density is substantially weaker (Weber et al., 2008 Cerebral Cortex). This dissociation suggests that regional CO variation more closely tracks sustained energetic demand and its vascular support than cell packing density. In addition, CO architecture closely matches the spatial distribution of NRF-2α, a transcription factor that regulates the expression of genes encoding CO, suggesting a tight coupling between metabolic architecture and transcriptional regulation (Guo et al., 2000 J. Comp. Neurol.; Nie and Wong-Riley 1999 J. Comp. Neurol.). More broadly, recent receptor autoradiography work has shown that neurotransmitter receptor expression varies systematically across macaque cortex, with a principal gradient of receptor density per neuron that tracks the cortical hierarchy from early sensory to higher cognitive areas (Froudist-Walsh et al., 2023 Nature Neuroscience). This indicates that receptor architecture is an important and tractable dimension of cortical specialization that could plausibly contribute to regional metabolic differences. However, because this receptor gradient primarily follows cortical hierarchy, it is likely to covary more closely with other features of visual system organization, such as receptive field size and SF tuning, than with eccentricity per se. As discussed above, SF tuning did not account for the present CO pattern as well as eccentricity did, and the darker staining observed in ML relative to early visual areas contrasts with the posterior-to-anterior increase in receptive field size across the visual hierarchy. Thus, although receptor architecture is clearly relevant to cortical specialization, the currently available receptor-gradient evidence does not alter our interpretation that eccentricity is the more informative axis in the present dataset.

We recognize that analogous vascular, transcriptional, and receptor data remain sparse in category-selective regions of the IT cortex. This limitation bears directly on interpretation: while CO provides a biologically grounded index of metabolic demand, it does not on its own distinguish among the cellular, vascular, or molecular mechanisms that give rise to regional metabolic specialization. As highlighted in recent work integrating *in vivo* functional localization with postmortem histology (e.g., Grimaldi et al., 2016 Neuron; Oishi et al., 2024 Cell Reports), additional measures such as cellular density, vascularization, receptor distributions, and transcriptional gradients will be essential for further mechanistic dissection.

Accordingly, we have added the biological basis of CO as a marker of metabolic architecture in the Introduction. We have revised the Discussion to clarify that our conclusions are grounded in CO’s established relationship to oxidative metabolism, while explicitly acknowledging that the mechanistic basis of metabolic differences in category-selective cortex remains an open question. In this sense, the present results define a constrained metabolic phenotype associated with eccentricity bias that future multimodal histological and molecular work can begin to explain.

**Specific changes:**

In the Introduction, we write (p. 3, lines 48–56):

Energy production in neurons is primarily mediated by mitochondria through oxidative phosphorylation (OXPHOS) complexes located in the inner mitochondrial membrane. Cytochrome oxidase (CO), which catalyzes the terminal step of OXPHOS, has long been used as a histochemical marker of sustained metabolic demand driven largely by synaptic processing. Several studies have demonstrated that CO staining changes transsynaptically following prolonged alterations in functional input and is preferentially associated with dendritic and postsynaptic metabolic demands [[4,5]](https://paperpile.com/c/rtlte2/X5OA+euTd). These findings indicate that regional differences in CO primarily reflect stable differences in metabolic architecture rather than transient stimulus-evoked activity.

In the Discussion, we write (pp. 18–19, lines 363–374):

Neuronal metabolism is supported by coordinated vascular supply and molecular regulatory mechanisms. In V1, CO intensity correlates tightly with microvascular density, whereas its correlation with neuronal density is substantially weaker [[27,28]](https://paperpile.com/c/rtlte2/ty8f+DVZb), suggesting that the observed regional variation in CO is not well explained by differences in cell packing density alone and instead more closely reflects differences in sustained energetic demand and its vascular support. Moreover, CO architecture has been shown to closely match the spatial distribution of NRF-2α, a transcription factor that regulates the expression of genes encoding CO, suggesting a tight coupling between metabolic architecture and transcriptional regulation [[29,30]](https://paperpile.com/c/rtlte2/6XMy+QilE). Whether similar relationships between CO and both vascular and transcriptional specializations hold in category-selective regions of IT and across the visual cortex remains to be determined. Multimodal integration of CO histology with quantitative measures of these architectures across the visual hierarchy will be important for further testing the basis of the CO gradients observed here.

4) There is an extensive literature, especially for human brain, in relation to gradient-wise organization (J.W. Vogel et al., 2024 and others). In my opinion, it is important for the Authors to briefly review this (in the context of their emphasis on "eccentricity").

We thank the reviewer for this suggestion and agree that gradient-wise organization has become an influential framework in contemporary neuroscience, particularly in the human brain. In response, we have revised the manuscript to briefly situate our findings in relation to this literature while also clarifying our terminology. Because the term gradient is used across the literature to describe organizational principles at multiple spatial and biological scales, we revised the manuscript to now consistently use the term topography when referring to retinotopic eccentricity. This change is intended to clearly distinguish sensory topographic organization from broader whole-brain gradient frameworks derived from functional connectivity, cortical microstructure, receptor distributions, or gene expression (e.g., Margulies et al., 2016 PNAS; Huntenburg et al., 2018 TiCS; Vogel et al., 2024 PNAS). References to gradient-based frameworks are retained where appropriate but are reserved for studies operating at these broader scales.

Within this clarified framework, retinotopic eccentricity represents a well-established and biologically grounded form of sensory topography, and one of the most extensively characterized principles of cortical organization (Kaas, 1997, Brain Res. Bull.). Retinotopic eccentricity has been mapped across early, intermediate, and higher-order visual cortex in both humans and macaques using convergent electrophysiological, anatomical, and fMRI approaches (e.g., Brewer et al., 2005 Nat. Neurosci.; Wandell et al., 2007 Neuron; Arcaro et al., 2009 J Neurosci.; Kolster et al., 2009 J Neurosci.; Janssens et al., 2014 J Neurosci.; Kolster et al., 2014 J Neurosci.). Work from our group and others has further demonstrated that eccentricity topography extends well beyond early visual cortex, shaping large-scale functional correlations, supra-areal clustering, and developmental proto-organization across the primate visual system (Hasson et al., 2002 Neuron; Arcaro et al., 2015 eLife; Arcaro et al., 2015 J Neurosci.; Arcaro and Livingstone, 2017 eLife). These findings directly motivated the use of eccentricity as an organizing axis in the present study and provide a systems- and developmentally grounded framework for interpreting the observed metabolic differences. Building on this framework, the present results show that CO intensity varies continuously along the eccentricity topography, rather than being restricted to discrete category-selective regions.

We recognize that recent gradient-based frameworks provide valuable unifying perspectives on cortical organization across modalities. While it is tempting to draw direct links between sensory topography and these broader organizational axes, such relationships remain relatively underexplored. To date, only a small number of studies have explicitly attempted to bridge these levels by relating sensory topography to large-scale intrinsic functional connectivity organization (e.g., Yeo et al., 2011 J Neurophysiol.; Buckner and Krienen, 2013 TiCS). Other work has demonstrated transcriptomic and microstructural gradients across visual cortex at broader hierarchical or areal scales (e.g., Gomez et al., 2019 Plos Biology; Gomez et al., 2021 Brain Struct Funct.), but direct links between such gradients and retinotopic eccentricity outside of early visual cortex remain limited.

Accordingly, in the present study we emphasize eccentricity as a canonical, evolutionarily conserved form of sensory topography that provides a concrete and well-validated organizing axis for interpreting cortical metabolic architecture, without assuming a direct correspondence to whole-brain functional or molecular gradient frameworks. We have revised the Discussion to reflect this framing and to situate our findings within both the long-standing retinotopy literature and contemporary gradient-based perspectives, while maintaining clear conceptual boundaries between these levels of organization.

**Specific changes:**

In the Discussion, we write (pp. 21–22, lines 431–440):

### **Broader implications for cortical organization**

Recent gradient-based frameworks have provided valuable unifying perspectives on cortical organization across modalities, including functional connectivity, microstructure, receptor distributions, and gene expression [[45,46]](https://paperpile.com/c/rtlte2/uvkb+Xm3B). Understanding how sensory topography relates to these broader organizational axes will be an important direction for future work, as direct links between them remain relatively underexplored and only limited studies have attempted to bridge sensory topography with large-scale intrinsic connectivity or molecular organization [[47–50]](https://paperpile.com/c/rtlte2/hDrX+Jvq2+BCTp+BoPh). In this context, we interpret our findings as demonstrating that cortical metabolic architecture varies systematically along a well-established sensory topographic axis, without assuming a direct correspondence to whole-brain functional or molecular gradient frameworks.

Reviewer #2: Oishi et al. combined cytochrome oxidase (CO) in 2 monkeys with fMRI to assess retinotopic maps and visual category selectivity in 9 monkeys. They found that CO indicates metabolic demands which vary with eccentricity (higher for central vision), resulting in a particularly expressed gradient in ML (middle lateral face patch) compared to LPP (lateral place patch).

To the best of my knowledge, these results are new and have the potential to uncover how higher-order representations arise in the visual cortical system. The manuscript is very well written. Since I am not a monkey researcher, I am not able to judge the anatomical methods.

The only major concern I have is the scare discussion, which seems to underuse the exceptional data and results reported in the Results section, see below.

Remark: Unfortunately, the PDF for download did not have page or line numbers. Thus, when referring to page numbers, I use those of the PDF file, which starts with a questionnaire.

We appreciate the Reviewer’s positive evaluation and constructive comments. We address each comment below, point by point. We apologize for the inconvenience. We include page numbers and line numbers in the revised manuscript file.

Page 11: ML and LPP are defined in the Results section but used, e.g., in the abstract earlier; better add definition to the abstract.

We thank the reviewer for this comment. We have now added a definition of ML and LPP to the Abstract.

**Specific changes:**

In the Abstract, we write (p. 2, lines 29–32):

We found that the middle lateral (ML) face-selective region, which is biased toward central vision, exhibits higher CO intensity than the lateral place patch (LPP), a scene-selective region biased toward peripheral vision.

Page 12: "These findings demonstrate that cortical metabolic architecture reflects functional layout, shaped by both the spatial structure of visual input and the processing demands of distinct visual domains." Are these independent aspects? It could be argued that the spatial structure is the result of the processing demands.

We thank the reviewer for raising this point. We completely agree that the spatial structure of visual input and the processing demands of visual domains are not independent. A large body of work suggests that the distribution of visual sampling is closely linked to the computational demands placed on the system, for example because central vision supports the processing required for tasks such as object and face recognition (e.g., Hasson et al., 2002 Neuron). We have therefore revised the sentence to avoid implying that these are distinct influences.

**Specific changes:**

In the Introduction (pp. 3–4), the revised sentence now reads:

These findings suggest that visual cortical metabolic architecture is shaped by the spatial structure of visual sampling and the processing demands that such sampling supports.

Page 13: Please add how face selectivity and scene selectivity were defined statistically.

We thank the reviewer for this suggestion. We have now clarified how probabilistic face-selective and scene-selective ROIs were defined statistically in the revised manuscript.

**Specific changes:**

In the Materials and Methods (p. 5, lines 94–96), we write:

In the generation of these atlases, face- and scene-selective voxels were defined as clusters of > 10 adjacent voxels that responded more strongly to images of faces compared to inanimate objects, or to images of scenes compared to images of other categories, respectively, as determined using a voxel-wise GLM (p < 0.0001, false discovery rate [FDR]-corrected). The probabilistic maps of the ML and LPP were created by calculating the percentage of subjects included in each voxel of NMT brain. For subsequent analyses, the probabilistic maps were thresholded at 70% to retain voxels with high inter-subject consistency.

Figure 3: please indicate the different scales for M1 and M2.

We thank the reviewer for pointing this out. The y-axis range (CO intensity level [A.U.]) and color scale (number of columnar bins) are different between M1 and M2. We have now explicitly indicated this in the figure caption to avoid any confusion.

**Specific changes:**

In the legend of the Figure 3 (p. 12, lines 231–233), we added the following statement:

Different CO intensity ranges and color scales were used for M1 and M2 to accommodate differences in CO signal range and the total number of columnar bins.

Minor suggestion: Figure 4: In the text results of the deep portion of the grey matter are discussed before those of the superficial portion. I would mirror this in Figure 4 (A-left: Deep, A-right: superficial)

We thank the reviewer for this suggestion. We reordered the panels such that the deep portion is shown on the left and the superficial portion on the right to match the order in which they are described in the text.

**Specific changes:**

We revised Figure 4 (p. 15):

**Fig. 4 CO intensity variation across occipitotemporal areas reflects eccentricity biases, with additional effects in face-selective regions and input layers of V1.**

Page 21: The idea that visual experience contributes to metabolic organization is in accord with studies in individuals with reversed congenital cataracts, who show a lower representation of the fovea (https://doi.org/10.1016/j.cub.2023.10.010), as well as reduced face selectivity in inferior temporal cortex (https://doi.org/10.1002/hbm.70316).

Please explain how developmental prosopagnosia might help to clarify how visual experience/ category experience shapes metabolic architecture. These individuals have, to the best of my knowledge, "typical" visual input from the eyes and no V1 deficits. Sight recovery individuals with a history of congenital blindness might be a more fruitful study approach in humans.

We thank the reviewer for this helpful suggestion and the specific references. Upon reflection, we agree that developmental prosopagnosia is not the most appropriate model for examining how visual experience shapes metabolic architecture. As the reviewer noted, individuals with developmental prosopagnosia typically have “typical” visual input, intact early visual cortical organization, and normal gaze patterns when viewing faces (Peterson et al., 2019 J. Vis.; Stehr et al., 2025 eLife). Their deficits do not provide a direct test of experience-dependent questions we raise in the Discussion.

The reviewer's suggestion to focus on sight recovery individuals is well taken. Individuals with reversed congenital cataracts experience early deprivation of patterned visual input and subsequently show both diminished foveal representation (Heitmann et al., 2023 Curr. Biol.) and reduced face selectivity in inferior temporal cortex despite restored vision (Rączy et al., 2025 Hum. Brain Mapp.). This population provides a more direct test of how early visual experience shapes the retinotopic and category-selective organization that our findings link to metabolic architecture.

We have revised the Discussion accordingly, removing developmental prosopagnosia and highlighting sight recovery individuals.

**Specific changes:**

In the revised Discussion (p. 21, lines 419–429), we now write:

Studies of humans with altered early visual experience offer valuable opportunities to test how experience shapes metabolic organization. Individuals with reversed congenital cataracts show diminished foveal representation [[43]](https://paperpile.com/c/rtlte2/ZCI2) and reduced face selectivity in inferior temporal cortex despite restored vision [[44]](https://paperpile.com/c/rtlte2/NZGu), suggesting long-lasting consequences of early visual deprivation for both retinotopic and category-selective organization. Examining metabolic architecture in such individuals could reveal whether the CO gradients we observe depend on typical early visual input. Together with experimental manipulations in animals, such as selective deprivation of specific visual features or training with novel stimuli [[18]](https://paperpile.com/c/rtlte2/0Xa2), these approaches could clarify how visual experience shapes cortical metabolic organization. Integrating CO histology with developmental and experiential manipulations will help clarify how these metabolic gradients arise and are maintained.

Discussion: is a bit scant and does not yet deals with all aspects of the results nor provides mechanistic explanations. For example, it is considered that cones are more energy demanding than rods. How does this translate to cortical neurons? Are neural compositions different for different parts of the inferior temporal cortex?

We thank the reviewer for this comment. We agree that the Discussion needed a clearer treatment of how retinal metabolic variability might be translated into cortical metabolic organization, as well as a more explicit discussion of the neural factors that could contribute to the CO differences we observe. We have expanded the Discussion to outline plausible mechanisms while acknowledging that our study is correlational and cannot directly test these mechanisms.

In the revised manuscript, we now discuss how retinotopic connectivity provides a potential route by which metabolic differences associated with cone-rich versus rod-rich retinal regions could propagate through the visual hierarchy and contribute to foveal biases in cortical metabolic architecture, consistent with classic demonstrations that CO expression reflects sustained, transsynaptic activity-dependent metabolic regulation (Wong-Riley and Welt, 1980 PNAS; Wong-Riley, 1989 TiCS). We further discuss evidence that, in V1, CO intensity correlates with microvascular density and with the spatial distribution of NRF-2α, suggesting that metabolic architecture is coupled to both vascular supply and transcriptional regulation (Weber et al., 2008 Cerebral Cortex; Guo et al. 2000 J. Comp. Neurol.; Nie and Wong-Riley 1999 J. Comp. Neurol.). At the same time, we now state more explicitly that whether comparable vascular, cellular, or molecular differences distinguish different parts of the inferotemporal cortex remains largely unknown.

**Specific changes:**

In the revised Discussion (pp. 17–18, lines 334–349), we now write:

### **Mechanisms underlying eccentricity-dependent metabolic gradients**

Our findings reveal a strong relationship between eccentricity and CO intensity. Why might cortical regions representing central vision be more metabolically demanding? One possibility is that neural circuits representing central visual space are specialized for higher acuity processing and finer spatial discrimination than those representing peripheral space, and that these circuit properties carry greater sustained energetic demands. In line with this idea, retinal cones dominate the fovea and support high-acuity vision, but they do so at a steep energetic cost [[20–22]](https://paperpile.com/c/rtlte2/x5MHB+TkAzw+57kxK). Their higher mitochondrial content, sustained ion exchange, and increased synaptic transmission result in elevated oxidative metabolism relative to peripheral rods [[23,24]](https://paperpile.com/c/rtlte2/mULok+c0aFr). The retinotopic organization of the visual cortex provides one plausible route by which such retinal differences could influence the metabolic organization across the visual hierarchy. Because retinotopic connectivity preserves spatial relationships from the retina through successive cortical stages, differences in metabolic demand associated with central versus peripheral vision could influence cortex, contributing to a foveal bias in cortical metabolic expenditure, consistent with classic demonstrations that cytochrome oxidase expression reflects sustained, transsynaptic activity-dependent metabolic regulation [[4,5]](https://paperpile.com/c/rtlte2/X5OA+euTd).

In the Introduction (p. 3, lines 48–56), we now write:

Energy production in neurons is primarily mediated by mitochondria through oxidative phosphorylation (OXPHOS) complexes located in the inner mitochondrial membrane. Cytochrome oxidase (CO), which catalyzes the terminal step of OXPHOS, has long been used as a histochemical marker of sustained metabolic demand driven largely by synaptic processing. Several studies have demonstrated that CO staining changes transsynaptically following prolonged alterations in functional input and is preferentially associated with dendritic and postsynaptic metabolic demands [[4,5]](https://paperpile.com/c/rtlte2/X5OA+euTd). These findings indicate that regional differences in CO primarily reflect stable differences in metabolic architecture rather than transient stimulus-evoked activity.

In the Discussion (pp. 18–19, lines 363–374), we now write:

Neuronal metabolism is supported by coordinated vascular supply and molecular regulatory mechanisms. In V1, CO intensity correlates tightly with microvascular density, whereas its correlation with neuronal density is substantially weaker [[27,28]](https://paperpile.com/c/rtlte2/ty8f+DVZb), suggesting that the observed regional variation in CO is not well explained by differences in cell packing density alone and instead more closely reflects differences in sustained energetic demand and its vascular support. Moreover, CO architecture has been shown to closely match the spatial distribution of NRF-2α, a transcription factor that regulates the expression of genes encoding CO, suggesting a tight coupling between metabolic architecture and transcriptional regulation [[29,30]](https://paperpile.com/c/rtlte2/6XMy+QilE). Whether similar relationships between CO and both vascular and transcriptional specializations hold in category-selective regions of IT and across the visual cortex remains to be determined. Multimodal integration of CO histology with quantitative measures of these architectures across the visual hierarchy will be important for further testing the basis of the CO gradients observed here.

The role of receptive field size is discussed. However, human fMRI studies have shown that these increase for face-selective regions in IT compared to early visual cortex; however, ML seems to be characterized by especially high CO gradients.

We thank the Reviewer for raising this point. We agree that absolute receptive field size increases from early visual cortex to inferotemporal cortex, and that face-selective regions in IT have larger receptive fields than neurons in V1. This indicates that absolute receptive field size alone cannot account for the metabolic gradients we observe (Fig 4A). We now consider this to be an important example of the dissociation between eccentricity and other widely known co-aligned functional representations.

In addition, we examined spatial frequency (SF), which covaries with eccentricity across the visual cortex (Srihasam et al. 2014, Nature Neuroscience) and is present early postnatally (Arcaro et al. 2017, eLife). SF therefore also provides a useful comparison to assess whether the observed CO pattern reflects eccentricity or another closely related visual dimension. We used the same registration pipeline to project group-average SF maps onto the histological sections. These SF maps were generated from fMRI data in four macaques using a previously published phase-encoded SF-mapping protocol (Arcaro and Livingstone, 2017 eLife). Across all occipitotemporal cortex bins, SF and CO were significantly negatively correlated in both monkeys (M1, Pearson’s r = -0.463; M2, r = -0.347; S4 FigA). However, these correlations were weaker than those observed between eccentricity and CO (M1, r = -0.572; M2, r = -0.441; Fig 3). In a multiple regression model with SF and eccentricity as predictors of CO, eccentricity accounted for more unique variance than SF, which accounted for relatively little unique variance (M1, partial R^2^ = 0.001 for SF and 0.143 for eccentricity, VIF = 2.620; M2, partial R^2^ = 0.001 for SF and 0.087 for eccentricity, VIF = 2.908; S2 TableA). Within category-selective regions, SF also showed weaker and less consistent relationships with CO than eccentricity did (face-selective region ML, Pearson’s r = -0.342, partial R^2^ = 0.004 for SF and 0.130 for eccentricity, VIF = 2.610; scene-selective region LPP, r = 0.105, partial R^2^ = 0.027 for SF and 0.281 for eccentricity, VIF = 1.004; all ps < 0.001; S4 FigB, S2 TableB). Together, these analyses indicate that SF also covaries with CO, but more weakly than eccentricity.

We have therefore revised the Discussion to clarify that receptive field size and SF may contribute to metabolic demand, but do not appear sufficient on their own to explain the observed pattern. Together, these considerations support the relevance of eccentricity for interpreting the observed metabolic gradients, without implying that eccentricity uniquely explains them.

**Specific changes:**

In the Results, we write (pp. 12–13, lines 237–252):

Because spatial frequency (SF) and eccentricity are known to covary across the visual cortex [[18,19]](https://paperpile.com/c/rtlte2/0Xa2+ixIY), we asked whether SF is also associated with CO. Across all occipitotemporal cortex bins, SF and CO were significantly negatively correlated in both monkeys (M1, Pearson’s r = -0.463; M2, r = -0.347; both ps < 0.001; S4 FigA). However, these correlations were weaker than those observed between eccentricity and CO (Fig 3). In a multiple regression model with SF and eccentricity as predictors of CO, eccentricity accounted for more unique variance than SF, which accounted for relatively little unique variance (M1, partial R^2^ = 0.001 for SF and 0.143 for eccentricity, VIF = 2.620; M2, partial R^2^ = 0.001 for SF and 0.087 for eccentricity, VIF = 2.908; S2 TableA). Within category-selective regions, SF also showed weaker and less consistent relationships with CO than eccentricity did (ML, Pearson’s r = -0.342 [p < 0.001], partial R^2^ = 0.004 for SF and 0.130 for eccentricity, VIF = 2.610; LPP, r = 0.105 [p < 0.001], partial R^2^ = 0.027 for SF and 0.291 for eccentricity, VIF = 1.004; S4 FigB, S2 TableB). Within LPP, the observed SF-CO correlation was small and opposite in sign to the negative correlations observed elsewhere, further suggesting that SF was not reliably associated with CO in this region. Together, these control analyses indicate that SF also covaries with CO, but more weakly than eccentricity, suggesting that eccentricity is more closely linked to the observed CO organization.

In the revised Discussion (p. 18, lines 350–362), we now write:

The CO-eccentricity relationship may also be shaped by multiple co-aligned factors. Eccentricity covaries with several functionally relevant properties, including SF tuning, receptive field size, curvature sensitivity, and category selectivity [23–26]. Accordingly, the observed CO gradients should not be interpreted as uniquely attributable to eccentricity. Consistent with this, SF also covaried with CO, but these relationships were weaker and less consistent than those observed for eccentricity (S4 Fig). Another feature closely related to eccentricity, receptive field size, also does not provide a sufficient account of the observed metabolic gradients. Absolute receptive field size increases along the visual hierarchy from early visual cortex to IT [[25,26]](https://paperpile.com/c/rtlte2/nsfH+O0HG), yet the face-selective region ML exhibited elevated CO levels relative to early visual areas, despite occupying a hierarchical position associated with larger receptive fields (Fig 4A). This dissociation further suggests that, although multiple co-aligned visual properties may contribute to the observed metabolic organization, eccentricity remains the more informative axis for interpreting the present CO pattern.

In the Materials and Methods, we write about the analysis using the probabilistic SF maps (pp 26–27, lines 552–566):

We used the same registration pipeline to project group-average spatial frequency (SF) maps onto the histological sections for control analyses. These SF maps were generated from fMRI data in four macaques using a previously published phase-encoded SF-mapping protocol [[19]](https://paperpile.com/c/rtlte2/ixIY). In that protocol, stimuli swept across eight logarithmically sampled SF values (3, 2, 1, 0.8, 0.5, 0.4, 0.2, and 0.1 cycles per degree [cpd]), and voxel-wise SF preferences were estimated using a Fourier-based analysis analogous to the retinotopy pipeline. SF preference maps were then averaged across subjects. To facilitate comparison with CO intensity, voxel-wise SF preferences were rescaled to a continuous range of 0–100 (where 0 corresponds to 3 cpd and 100 corresponds to 0.1 cpd) using a logarithmic transform that preserves the relative spacing of the sampled SF values. We restricted the analysis to voxels with a group-average F-statistic greater than 5. For the present study, we projected the group-average SF maps onto the reconstructed histological volume using the same nonlinear registration procedure used for the eccentricity and visual-area maps. Columnar bins that overlapped with the eccentricity maps were used for correlation analyses and for multiple regression analyses. In the regression analyses, SF and eccentricity served as predictors of CO.

In the Supplementary Information Legends (p. 38, lines 807–815 and lines 835–840):

**S4 Fig. Spatial frequency (SF) covaries with CO, but more weakly and less consistently than eccentricity. (A)** SF-CO correlations across occipitotemporal cortex. The inset image shows a group average SF map (n = 4) shown in the same surface view as Fig 2A. The color code indicates the preferred SF at each cortical location, covering a continuous range of 0–100 (where 0 corresponds to 3 cpd and 100 corresponds to 0.1 cpd). Two-dimensional histograms show the distribution of spatial bins across occipitotemporal cortex as a function of spatial frequency (bin width = 2 [A.U.], matched to the relative bin width used for eccentricity in Fig 3) and of CO intensity (bin width = 0.01) for monkey M1 (left) and M2 (right). Conventions are as in Fig 3. **(B)** SF-CO correlations within ML (left) and LPP (right). Conventions are as in Fig 2D. Pearson’s r is indicated in each panel (all ps < 0.001).

**S2 Table. Multiple linear regression results with spatial frequency and eccentricity as predictors of CO.** Multiple linear regressions were performed across all occipitotemporal cortex bins in both monkeys (A) and within face patch ML and place patch LPP (B). SE, standard error; CI, confidence interval; RMSE, root mean squared error; VIF, variance inflation factor. Partial R^2^ indicates the unique variance explained by each variable after accounting for the other predictor. VIF was used to assess multicollinearity.

The results of Figure 4 are not discussed at all. Why are deviations from the average retinotopic - CO relation different for different cortical layers, and why does this interact with ROI (e.g., V1 vs. ML)? This finding seems to be crucial and might have the potential to make a significant contribution to our understanding of how higher-order representations emerge in the visual system.

We thank the reviewer for highlighting this point and agree that the depth-specific deviations shown in Figure 4 represent a key finding that warrants explicit discussion. While the Results describe this depth-dependent dissociation, with V1 showing elevated CO in deeper portions and ML showing elevated CO in more superficial portions, we acknowledge that the original manuscript did not sufficiently interpret these patterns or explain why they differ across regions.

We have now expanded the Discussion to address this directly. In V1, elevated CO in deeper portions is consistent with the high metabolic demands of layer IVc, which receives dense thalamic input from the LGN. In contrast, the strongest deviations in ML occur in more superficial portions, suggesting a different source of metabolic demand. In the extrastriate cortex, superficial portions are more strongly associated with feedback projections, lateral corticocortical connectivity, and other forms of local recurrent processing. Anatomical tracing has further shown that ML has particularly dense corticocortical connectivity with other face-processing regions and visual areas within the face network (Grimaldi et al., 2016 Neuron), which could contribute to elevated metabolic demand in superficial portions.

This interpretation is also consistent with our prior finding that ML exhibits especially pronounced CO staining in superficial portions compared to other face patches (Oishi et al., 2024 Cell Reports). At the same time, we emphasize that the present data are descriptive, and whether these depth-specific metabolic signatures reflect differences in input sources, local circuit properties, or processing demands remains an open question. Nevertheless, the contrast between V1 and ML suggests that depth-resolved CO analysis may help reveal how laminar and circuit-level organization contribute to functional specialization in higher-order visual cortex.

For comparison, we also reproduce a figure from our prior publication (Oishi et al., 2024 Cell Reports) that illustrates the distinct depth-dependent CO staining profiles of V1 and ML in greater detail (Reviewer Figure).

 **Reviewer Figure** (reproduction of Figure 2 from Oishi et al. 2024, Cell Reports).　**Architectonic profiles of fMRI-defined V1 and face patch ML from histological data in the same individual**
(A) Primary visual cortex (V1) and ML localized in the same histological section as shown in Figure 1B, with magnifications of V1 (pink boxes) and ML (orange boxes). Dashed colored lines illustrate the definitions of V1 and ML from fMRI data. (B) Cortical depth maps of V1 (pink) and surrounding cortex. (C) CO depth profiles across V1 sections. The pale pink curves illustrate CO profiles of each section, and the bolded pink curve illustrates the mean staining across sections. The black curve shows the depth profile for the section shown in (A). Higher numbers indicate darker staining intensity. The peak of the CO staining at mid-cortical depths (black arrow) corresponds to the visible thick band in V1 (A, black arrow). (D) Cortical depth map of ML (orange) and surrounding cortex. Conventions are consistent with (B). (E) CO staining across ML sections (n = 11). The pale orange curves illustrate the CO intensity of each section, and the bolded orange curve illustrates the mean intensity across sections. The black curve shows the depth profile for the section shown in (A). The dark staining toward mid-depths (red arrow) corresponds to the visible band of dark staining in ML (A, red arrow).

**Specific changes:**

In the revised Discussion (p. 20, lines 392–401), we now write:

### **Region-specific CO architectures**

Our depth-resolved residual analyses revealed region-specific depth signatures (Fig 4). V1 showed darker CO staining in deeper portions, consistent with the dense thalamic input to layer IVc [[37]](https://paperpile.com/c/rtlte2/QQz6). In contrast, ML showed the darkest CO staining in more superficial portions. This pattern aligns with prior findings of pronounced CO staining in superficial portions of ML [[2]](https://paperpile.com/c/rtlte2/W8cF) and with anatomical evidence for dense corticocortical connectivity of this region with other face-processing regions [[38]](https://paperpile.com/c/rtlte2/xXit). Whether these depth-specific CO signatures reflect differences in input sources, local circuit properties, or processing demands remains an open question, but they highlight the potential of depth-resolved CO analysis to reveal how laminar and circuit-level organization contributes to functional specialization in higher-order visual cortex.

Nice prospects are given for the dorsal stream, but any concrete hypothesis? The discussion is a bit decoupled from the abstract and main conclusion. I might have missed direct comparisons between regions, e.g., between ML and LPP.

We thank the reviewer for this comment and agree that the discussion of the dorsal stream should be more tightly anchored to the paper’s main conclusions. Our primary comparison was between the face-selective region ML and the scene-selective region LPP, in order to test whether differences in metabolic architecture between ventral temporal regions could be understood in relation to retinotopic eccentricity. We agree, however, that the broader implication of this framework is whether the observed CO-eccentricity relationship may generalize beyond the ventral cortex.

Our data suggest a concrete hypothesis that CO intensity across both dorsal and ventral visual cortex is organized primarily by retinotopic eccentricity. Under this view, regions with more central visual field biases should exhibit darker CO staining than regions with more peripheral biases, regardless of whether they belong to the ventral or dorsal stream. Although dorsal and ventral streams differ in magnocellular and parvocellular input biases, temporal dynamics, contrast sensitivity, and developmental trajectories (Livingstone and Hubel 1988 Science; Stigliani et al. 2017 PNAS; Ayzenberg and Arcaro 2026 Neuron in press; Bourne and Rosa 2006 Cerebral Cortex), they also differ systematically in retinotopic bias, with ventral regions tending to be more foveally biased (e.g., Janssens et al. 2014 J Neurosci.) and dorsal regions more peripherally biased (e.g., Arcaro et al. 2011 J Neurosci.; Rima et al. 2020 Brain Struct Funct.). This raises the possibility that apparent metabolic differences between streams may reflect differences in retinotopic sampling. Consistent with this possibility, MT, a canonical magnocellular-recipient dorsal-stream area, falls along the same CO-eccentricity relationship as ventral-stream regions in our dataset (Fig 4A). Together, these observations suggest that metabolic organization may be structured by eccentricity across the visual cortex, with stream-level differences emerging largely from differences in retinotopic bias.

We have revised the Discussion accordingly to clarify that this observation supports the paper’s broader conclusion that cortical metabolic architecture reflects the spatial structure of visual input across regions. It also motivates a specific prediction for future work, that dorsal-stream regions with central versus peripheral visual field biases should show corresponding differences in CO intensity.

**Specific changes:**

In the revised Discussion (p. 22, lines 441–455), we now write:

Our primary comparison was between the face-selective region ML and the scene-selective region LPP, in order to test whether differences in metabolic architecture between ventral temporal regions could be understood in relation to retinotopic eccentricity. A broader implication of this framework is that the CO-eccentricity relationship may generalize beyond the ventral temporal cortex to other retinotopically organized visual regions, including the dorsal stream. Because dorsal and ventral regions differ systematically in magnocellular and parvocellular input biases, temporal dynamics, contrast sensitivity, and developmental trajectories [[40,51–53]](https://paperpile.com/c/rtlte2/5JCnf+KgcMV+K9eZg+z80Qw), metabolic organization may also differ between streams. At the same time, ventral regions tend to be more foveally biased, whereas dorsal regions tend to be more peripherally biased [[12,54,55]](https://paperpile.com/c/rtlte2/0SV4+0kzZ+T1g5). Thus, any average metabolic differences between streams may reflect differences in retinotopic connectivity rather than stream identity itself. Consistent with this possibility, MT, a magnocellular-recipient dorsal-stream area, fell along the same CO-eccentricity relationship as ventral-stream regions (Fig 4A), suggesting that metabolic organization may vary with retinotopic eccentricity throughout visual cortex. Testing whether parietal regions with different eccentricity biases exhibit corresponding differences in CO intensity would provide a direct test of this hypothesis.

Questions: Were the 2 monkeys on which CO histology was performed among the monkeys for which fMRI retinotopy and categorical organization in the inferior temporal cortex were assessed? I would add an explicit statement to the text.

We thank the reviewer for raising this important clarification. Although the histology monkeys had individual face-patch localizations available from our prior work (Oishi et al., 2024, Cell Reports), they were not part of the independent fMRI datasets used to generate the probabilistic place-patch or retinotopy maps, and comparable individual localizations were therefore not available for place and eccentricity maps. To keep comparisons across face, place, and retinotopic ROIs methodologically consistent, we therefore used probabilistic atlases for all analyses. These atlases were derived from independent groups of monkeys (n = 6 for face patches, n = 9 for place patches, n = 6 for retinotopy) and aligned to each histological volume via registration to the NMT template brain.

Our prior study supported this approach by showing that, in the same animals, probabilistic face-patch definitions preserved the key qualitative CO patterns observed with individually defined patches, although with reduced effect sizes (Oishi et al., 2024, Cell Reports). To further address the concern regarding cross-animal alignment in the present study, we repeated the CO-eccentricity analysis using each individual monkey retinotopy map separately rather than the probabilistic eccentricity map (S3 Fig). The relationship remained consistently negative across all individual maps (all Pearson’s r ≤ −0.323), although somewhat weaker than with the group average, as expected for noisier single-subject estimates. Together, these analyses support the conclusion that the reported CO-eccentricity correspondence is not dependent on unusually precise atlas alignment and can be recovered despite the reduced precision of cross-animal mapping.

We have added an explicit statement to the Methods clarifying the relationship between the fMRI and histology samples.

**Specific changes:**

In the revised Materials and Methods (p. 25, lines 521–530), we now write:

To define the face- and scene-selective regions in the IT cortex, as well as retinotopic organization, we used a probabilistic group atlas generated from independent fMRI datasets (n = 6 for face patches, n = 9 for place patches, n = 6 for retinotopy). Although the histology monkeys had individual face-patch localizations available from our prior work [[2]](https://paperpile.com/c/rtlte2/W8cF), they were not part of the independent fMRI datasets used to generate the probabilistic place-patch or retinotopy maps. Comparable individual localizations were therefore not available for place-patch or retinotopic ROIs. Atlas-based definitions were therefore used throughout so that face, place, and retinotopic comparisons were performed under the same analysis constraints across ROIs. In our prior study, probabilistic face-patch definitions preserved the key qualitative CO staining patterns observed with individually defined face patches, although with reduced effect sizes [[2]](https://paperpile.com/c/rtlte2/W8cF).

In the Supplementary Information Legends (p. 38, lines 807–815):

**S3 Fig. CO–eccentricity correlations computed using eccentricity values from each individual fMRI subject (N = 6) were consistently negative.** Filled circles denote correlations obtained using individual eccentricity maps (N = 6), whereas the open circle denotes the correlation obtained using the probabilistic eccentricity map. **(A)** Correlations across the occipitotemporal visual cortex in M1 (left) and M2 (right), related to Fig 3. **(B)** Correlations across the occipitotemporal visual cortex in deep (left) and superficial (right) depth portions, related to Fig 4A. The correlations consistently deviated from zero (all Pearson’s r ≤ −0.323). This convergence supports the robustness of the CO-eccentricity relationship despite the lack of same-animal physiological mapping.

Reviewer #3: This is an interesting manuscript that examined the relationship between fMRI measures of eccentricity with cytochrome oxidase staining as an indicator of metabolism. The presentation is concise and in general the study is very well presented and written.

We thank the reviewer for this summary highlighting the contributions and overall positive assessment of our work.

A general overview of previous work on this area on primary sensory systems is likely necessary to catch up the reviewer on details of cytochrome oxidase and in vivo metabolism measurements. As is, the work reads like this is a new study without appropriate recognition of prior work (2DG results and/or other imaging results from PET or MRI would be very helpful).

This work could benefit from short-reaching extensions, even if brief, like comparing the CO staining with depth with mapping signals of eccentricity (amplitude) even if those are biased to the superficial layers.

We thank the reviewer for this constructive feedback. We agree that the manuscript would benefit from more explicit recognition of prior work on cytochrome oxidase and other approaches to measuring metabolic activity in the sensory cortex. In response, we have expanded the Introduction to clarify the cellular and metabolic basis of CO staining, and we have expanded the Discussion to situate the present findings in relation to prior work using classical 2-deoxyglucose methods as well as PET and MRI based approaches to metabolic mapping.

We also agree that relating depth-resolved CO staining to functional measures of eccentricity is, in principle, an interesting direction. However, a direct comparison between CO depth profiles and *in vivo* fMRI response amplitude is not feasible in the present dataset for methodological reasons. The eccentricity mapping data were acquired using uncalibrated MION fMRI, for which response amplitude primarily reflects stimulus-evoked changes in cerebral blood volume and is strongly influenced by baseline vascular properties and depth-dependent vascular biases, including a pronounced bias toward superficial cortical portions. In addition, the spatial distribution of fMRI response amplitude is affected by inhomogeneity in signal-to-noise ratio arising from factors such as coil placement, multichannel receive sensitivity, and slice acceleration, making amplitude difficult to interpret as a stable quantitative measure across cortical locations. Because CO staining indexes sustained oxidative metabolic capacity integrated over long timescales, a direct comparison between CO depth profiles and MION eccentricity-mapping amplitude would therefore conflate transient hemodynamic responses with long-term metabolic architecture.

Although a direct comparison to eccentricity-mapping amplitude is not interpretable in the present dataset, we performed a related depth-resolved analysis to ask whether the observed CO-eccentricity relationship was confined to a limited depth range. Specifically, we separated superficial and deep cortical portions to determine whether regional effects were driven by a restricted depth range or extended across cortical depth. Importantly, because eccentricity values are derived from the cortical surface and do not vary with depth, this analysis does not test laminar specificity of functional responses. The CO-eccentricity relationship was evident in both superficial and deep portions within face- and scene-selective patches (S1 Fig, related to Fig 2), as well as across the broader occipitotemporal visual cortex (S2 Fig, related to Fig 3). These findings indicate that the observed effects are not confined to a limited depth range but instead extend across cortical depth.

**Specific changes:**

In the revised Introduction (p. 3, lines 48–56), we now write:

Energy production in neurons is primarily mediated by mitochondria through oxidative phosphorylation (OXPHOS) complexes located in the inner mitochondrial membrane. Cytochrome oxidase (CO), which catalyzes the terminal step of OXPHOS, has long been used as a histochemical marker of sustained metabolic demand driven largely by synaptic processing. Several studies have demonstrated that CO staining changes transsynaptically following prolonged alterations in functional input and is preferentially associated with dendritic and postsynaptic metabolic demands [[4,5]](https://paperpile.com/c/rtlte2/X5OA+euTd). These findings indicate that regional differences in CO primarily reflect stable differences in metabolic architecture rather than transient stimulus-evoked activity.

In the revised Discussion (p. 19, lines 376–390), we now write:

**CO as an index of metabolic architecture**

The relationship between cortical metabolic architecture and functional organization has long been studied using a variety of metabolic mapping approaches in addition to CO histology. Classical histological studies using the 2-deoxyglucose (2-DG) method demonstrated spatially structured patterns of glucose utilization that closely correspond to functional architecture in visual cortex [[31–34]](https://paperpile.com/c/rtlte2/b2F0+NwMP+2gxO+nBgB). More recently, positron emission tomography (PET) using ^18^F-fluorodeoxyglucose (FDG) has enabled measurements of metabolic activity *in vivo* [[32]](https://paperpile.com/c/rtlte2/NwMP), and calibrated-fMRI approaches that estimate cerebral metabolic rate of oxygen (CMRO₂) can non-invasively probe metabolic activity [[35,36]](https://paperpile.com/c/rtlte2/0sz6+rQfL). However, these methods primarily capture short-term metabolic responses associated with stimulus-evoked or ongoing neural activity. By contrast, CO histochemistry indexes sustained oxidative metabolic capacity and has been shown in the early sensory cortex to align with stable features of functional organization. In this context, our findings suggest that metabolic architecture also tracks large-scale functional organization across the visual hierarchy, making CO histochemistry particularly well suited for studying stable cortical metabolic architecture.

In the Supplementary Information Legends (p. 38, lines 799–806):

**S1 Fig. CO-eccentricity correlations in face- and scene-selective regions across cortical depth.** Correlations are shown for ML (left) and LPP (right), with superficial portions shown in the top row and deep portions shown in the bottom row**.** Conventions are as in Fig 2D.

**S2 Fig. CO-eccentricity correlations across the occipitotemporal visual cortex are robust across cortical depth.** Each panel represents a two-dimensional histogram with linear-regression and low-order polynomial fits (red lines and gray curves, respectively). Pearson’s r is indicated in each panel. Conventions are as in Fig 3. Significant correlations are observed in both superficial (upper) and deep (lower) depth portions in M1 (left) and M2 (right).

Another possibility is discussing the relationship between CO staining and vascular or capillary density as these are likely linked. Other than that, I agree that evaluating metabolic markers with function remains an important understudied aspect in brain imaging research.

We thank the reviewer for this insightful suggestion and agree that the relationship between CO staining and vascular density is an important consideration. CO reflects oxidative metabolic capacity, and regions with elevated metabolic demand necessarily require corresponding vascular support to supply oxygen and glucose. Thus, the relationship between CO and vascular architecture is important for interpreting whether the CO patterns observed in our study reflect cellular metabolic demand, vascular supply, or coordinated scaling of both.

In the macaque visual system, a close correspondence between CO activity and microvascular density has been demonstrated in the primary visual cortex (Weber et al., 2008 Cerebral Cortex), supporting the use of CO staining as an index of steady-state metabolic demand. These findings indicate that metabolic architecture and vascular architecture are tightly co-organized in the early visual cortex. However, whether similar relationships extend to extrastriate and inferotemporal cortex, particularly within category-selective regions, remains largely unexplored, as comparable quantitative mapping of microvascular density, especially in conjunction with functional maps, has not yet been systematically performed.

**Specific changes:**

In the Discussion (pp. 18–19, lines 363–374), we now write:

Neuronal metabolism is supported by coordinated vascular supply and molecular regulatory mechanisms. In V1, CO intensity correlates tightly with microvascular density, whereas its correlation with neuronal density is substantially weaker [[27,28]](https://paperpile.com/c/rtlte2/ty8f+DVZb), suggesting that the observed regional variation in CO is not well explained by differences in cell packing density alone and instead more closely reflects differences in sustained energetic demand and its vascular support. Moreover, CO architecture has been shown to closely match the spatial distribution of NRF-2α, a transcription factor that regulates the expression of genes encoding CO, suggesting a tight coupling between metabolic architecture and transcriptional regulation [[29,30]](https://paperpile.com/c/rtlte2/6XMy+QilE). Whether similar relationships between CO and both vascular and transcriptional specializations hold in category-selective regions of IT and across the visual cortex remains to be determined. Multimodal integration of CO histology with quantitative measures of these architectures across the visual hierarchy will be important for further testing the basis of the CO gradients observed here.

**References (Response to Reviewers)**

[Arcaro, Michael J., Christopher J. Honey, Ryan E. B. Mruczek, Sabine Kastner, and Uri Hasson. 2015. “Widespread Correlation Patterns of fMRI Signal across Visual Cortex Reflect Eccentricity Organization.” *eLife* 4 (February). https://doi.org/](http://paperpile.com/b/SZqQGw/gSQ24)[10.7554/eLife.03952](http://dx.doi.org/10.7554/eLife.03952)[.](http://paperpile.com/b/SZqQGw/gSQ24)

[Arcaro, Michael J., and Margaret S. Livingstone. 2017a. “A Hierarchical, Retinotopic Proto-Organization of the Primate Visual System at Birth.” *eLife* 6 (July). https://doi.org/](http://paperpile.com/b/SZqQGw/mJKQu)[10.7554/eLife.26196](http://dx.doi.org/10.7554/eLife.26196)[.](http://paperpile.com/b/SZqQGw/mJKQu)

[Arcaro, Michael J., and Margaret S. Livingstone. 2017b. “Retinotopic Organization of Scene Areas in Macaque Inferior Temporal Cortex.” *The Journal of Neuroscience: The Official Journal of the Society for Neuroscience* 37 (31): 7373–7389.](http://paperpile.com/b/SZqQGw/Epc5x)

[Arcaro, Michael J., and Margaret S. Livingstone. 2021. “On the Relationship between Maps and Domains in Inferotemporal Cortex.” *Nature Reviews. Neuroscience* 22 (9): 573–583.](http://paperpile.com/b/SZqQGw/mEAwa)

[Arcaro, Michael J., Theodora Mautz, Vladimir K. Berezovskii, and Margaret S. Livingstone. 2020. “Anatomical Correlates of Face Patches in Macaque Inferotemporal Cortex.” *Proceedings of the National Academy of Sciences of the United States of America* 117 (51): 32667–32678.](http://paperpile.com/b/SZqQGw/0n8ZK)

[Arcaro, Michael J., Stephanie A. McMains, Benjamin D. Singer, and Sabine Kastner. 2009. “Retinotopic Organization of Human Ventral Visual Cortex.” *The Journal of Neuroscience: The Official Journal of the Society for Neuroscience* 29 (34): 10638–10652.](http://paperpile.com/b/SZqQGw/0v8Vt)

[Arcaro, Michael J., Mark A. Pinsk, and Sabine Kastner. 2015. “The Anatomical and Functional Organization of the Human Visual Pulvinar.” *The Journal of Neuroscience: The Official Journal of the Society for Neuroscience* 35 (27): 9848–9871.](http://paperpile.com/b/SZqQGw/BZY8K)

[Arcaro, Michael J., Mark A. Pinsk, Xin Li, and Sabine Kastner. 2011. “Visuotopic Organization of Macaque Posterior Parietal Cortex: A Functional Magnetic Resonance Imaging Study.” *The Journal of Neuroscience: The Official Journal of the Society for Neuroscience* 31 (6): 2064–2078.](http://paperpile.com/b/SZqQGw/OpRPi)

[Arcaro, Michael, and Margaret Livingstone. 2024. “A Whole-Brain Topographic Ontology.” *Annual Review of Neuroscience* 47 (1): 21–40.](http://paperpile.com/b/SZqQGw/2ZCmw)

[Ayzenberg, Vladislav, and Michael Arcaro. 2025. “Functional Organization of the Human Visual System at Birth and across Late Gestation.” In *Neuroscience*, No. Biorxiv;2025.09.22.677834v2. BioRxiv, September 22.](http://paperpile.com/b/SZqQGw/ejuMZ) <https://www.biorxiv.org/content/10.1101/2025.09.22.677834v1>[.](http://paperpile.com/b/SZqQGw/ejuMZ)

[Berntson, A., and W. R. Taylor. 2000. “Response Characteristics and Receptive Field Widths of on-Bipolar Cells in the Mouse Retina.” *The Journal of Physiology* 524 Pt 3 (May): 879–889.](http://paperpile.com/b/SZqQGw/nim2x)

[Bohraus, Yvette, Hellmut Merkle, Nikos K. Logothetis, and Jozien Goense. 2023. “Laminar Differences in Functional Oxygen Metabolism in Monkey Visual Cortex Measured with Calibrated fMRI.” *Cell Reports* 42 (11): 113341.](http://paperpile.com/b/SZqQGw/SR3c0)

[Bourne, James A., and Marcello G. P. Rosa. 2006. “Hierarchical Development of the Primate Visual Cortex, as Revealed by Neurofilament Immunoreactivity: Early Maturation of the Middle Temporal Area (MT).” *Cerebral Cortex (New York, N.Y.: 1991)* 16 (3): 405–414.](http://paperpile.com/b/SZqQGw/qFhQv)

[Brewer, Alyssa A., Junjie Liu, Alex R. Wade, and Brian A. Wandell. 2005. “Visual Field Maps and Stimulus Selectivity in Human Ventral Occipital Cortex.” *Nature Neuroscience* 8 (8): 1102–1109.](http://paperpile.com/b/SZqQGw/Kkjlb)

[Buckner, Randy L., and Fenna M. Krienen. 2013. “The Evolution of Distributed Association Networks in the Human Brain.” *Trends in Cognitive Sciences* 17 (12): 648–665.](http://paperpile.com/b/SZqQGw/eBlFB)

[Curcio, C. A., K. R. Sloan, R. E. Kalina, and A. E. Hendrickson. 1990. “Human Photoreceptor Topography.” *The Journal of Comparative Neurology* 292 (4): 497–523.](http://paperpile.com/b/SZqQGw/fhfYr)

[Duffy, Kevin R., and Margaret S. Livingstone. 2003. “Distribution of Non-Phosphorylated Neurofilament in Squirrel Monkey V1 Is Complementary to the Pattern of Cytochrome-Oxidase Blobs.” *Cerebral Cortex (New York, N.Y.: 1991)* 13 (7): 722–727.](http://paperpile.com/b/SZqQGw/rQpl5)

[Froudist-Walsh, Sean, Ting Xu, Meiqi Niu, et al. 2023. “Gradients of Neurotransmitter Receptor Expression in the Macaque Cortex.” *Nature Neuroscience* 26 (7): 1281–1294.](http://paperpile.com/b/SZqQGw/4Ylth)

[Gomez, Jesse, Zonglei Zhen, and Kevin S. Weiner. 2019. “Human Visual Cortex Is Organized along Two Genetically Opposed Hierarchical Gradients with Unique Developmental and Evolutionary Origins.” *PLoS Biology* 17 (7): e3000362.](http://paperpile.com/b/SZqQGw/xq4PA)

[Gomez, Jesse, Zonglei Zhen, and Kevin S. Weiner. 2021. “The Relationship between Transcription and Eccentricity in Human V1.” *Brain Structure & Function* 226 (9): 2807–2818.](http://paperpile.com/b/SZqQGw/g5Qpa)

[Grill-Spector, Kalanit, and Kevin S. Weiner. 2014. “The Functional Architecture of the Ventral Temporal Cortex and Its Role in Categorization.” *Nature Reviews. Neuroscience* 15 (8): 536–548.](http://paperpile.com/b/SZqQGw/BABtW)

[Grimaldi, Piercesare, Kadharbatcha S. Saleem, and Doris Tsao. 2016. “Anatomical Connections of the Functionally Defined ‘Face Patches’ in the Macaque Monkey.” *Neuron* 90 (6): 1325–1342.](http://paperpile.com/b/SZqQGw/mK1I6)

[Guo, Aili, Feng Nie, and Margaret Wong-Riley. 2000. “Human Nuclear Respiratory Factor 2α Subunit cDNA: Isolation, Subcloning, Sequencing, and in Situ Hybridization of Transcripts in Normal and Monocularly Deprived Macaque Visual System.” *Journal of Comparative Neurology* 417: 221–232.](http://paperpile.com/b/SZqQGw/RHP69)

[Hasson, Uri, Ifat Levy, Marlene Behrmann, Talma Hendler, and Rafael Malach. 2002. “Eccentricity Bias as an Organizing Principle for Human High-Order Object Areas.” *Neuron* 34 (3): 479–490.](http://paperpile.com/b/SZqQGw/lKAVx)

[Heitmann, Carolin, Minye Zhan, Madita Linke, et al. 2023. “Early Visual Experience Refines the Retinotopic Organization within and across Visual Cortical Regions.” *Current Biology* 33 (22): 4950–4959.e4.](http://paperpile.com/b/SZqQGw/GgZ6q)

[Huntenburg, Julia M., Pierre-Louis Bazin, and Daniel S. Margulies. 2018. “Large-Scale Gradients in Human Cortical Organization.” *Trends in Cognitive Sciences* 22 (1): 21–31.](http://paperpile.com/b/SZqQGw/RSxj)

[Ingram, Norianne T., Gordon L. Fain, and Alapakkam P. Sampath. 2020. “Elevated Energy Requirement of Cone Photoreceptors.” *Proceedings of the National Academy of Sciences of the United States of America* 117 (32): 19599–19603.](http://paperpile.com/b/SZqQGw/GZ2U7)

[Janssens, Thomas, Qi Zhu, Ivo D. Popivanov, and Wim Vanduffel. 2014. “Probabilistic and Single-Subject Retinotopic Maps Reveal the Topographic Organization of Face Patches in the Macaque Cortex.” *The Journal of Neuroscience: The Official Journal of the Society for Neuroscience* 34 (31): 10156–10167.](http://paperpile.com/b/SZqQGw/EOQSc)

[Kaas, J. H. 1997. “Topographic Maps Are Fundamental to Sensory Processing.” *Brain Research Bulletin* 44 (2): 107–112.](http://paperpile.com/b/SZqQGw/DxyIg)

[Kageyama, G. H., and M. T. Wong-Riley. 1984. “The Histochemical Localization of Cytochrome Oxidase in the Retina and Lateral Geniculate Nucleus of the Ferret, Cat, and Monkey, with Particular Reference to Retinal Mosaics and ON/OFF-Center Visual Channels.” *The Journal of Neuroscience: The Official Journal of the Society for Neuroscience* 4 (10): 2445–2459.](http://paperpile.com/b/SZqQGw/b6vF3)

[Kay, Kendrick N., Kevin S. Weiner, and Kalanit Grill-Spector. 2015. “Attention Reduces Spatial Uncertainty in Human Ventral Temporal Cortex.” *Current Biology* 25 (5): 595–600.](http://paperpile.com/b/SZqQGw/5L15U)

[Keller, Anna Lena, Almut Schüz, Nikos K. Logothetis, and Bruno Weber. 2011. “Vascularization of Cytochrome Oxidase-Rich Blobs in the Primary Visual Cortex of Squirrel and Macaque Monkeys.” *The Journal of Neuroscience: The Official Journal of the Society for Neuroscience* 31 (4): 1246–1253.](http://paperpile.com/b/SZqQGw/SuwWj)

[Kennedy, C., M. H. Des Rosiers, O. Sakurada, et al. 1976. “Metabolic Mapping of the Primary Visual System of the Monkey by Means of the Autoradiographic [14C]deoxyglucose Technique.” *Proceedings of the National Academy of Sciences of the United States of America* 73 (11): 4230–4234.](http://paperpile.com/b/SZqQGw/wSbn4)

[Kolster, Hauke, Thomas Janssens, Guy A. Orban, and Wim Vanduffel. 2014. “The Retinotopic Organization of Macaque Occipitotemporal Cortex Anterior to V4 and Caudoventral to the Middle Temporal (MT) Cluster.” *The Journal of Neuroscience: The Official Journal of the Society for Neuroscience* 34 (31): 10168–10191.](http://paperpile.com/b/SZqQGw/ho6ot)

[Kolster, Hauke, Joseph B. Mandeville, John T. Arsenault, Leeland B. Ekstrom, Lawrence L. Wald, and Wim Vanduffel. 2009. “Visual Field Map Clusters in Macaque Extrastriate Visual Cortex.” *The Journal of Neuroscience: The Official Journal of the Society for Neuroscience* 29 (21): 7031–7039.](http://paperpile.com/b/SZqQGw/kcjRP)

[Kornblith, Simon, Xueqi Cheng, Shay Ohayon, and Doris Y. Tsao. 2013. “A Network for Scene Processing in the Macaque Temporal Lobe.” *Neuron* 79 (4): 766–781.](http://paperpile.com/b/SZqQGw/1eTYO)

[Kravitz, Dwight J., Kadharbatcha S. Saleem, Chris I. Baker, Leslie G. Ungerleider, and Mortimer Mishkin. 2013. “The Ventral Visual Pathway: An Expanded Neural Framework for the Processing of Object Quality.” *Trends in Cognitive Sciences* 17 (1): 26–49.](http://paperpile.com/b/SZqQGw/9iVwH)

[Kushner, M. J., A. Rosenquist, A. Alavi, et al. 1988. “Cerebral Metabolism and Patterned Visual Stimulation.” *Neurology* 38 (1): 89–89.](http://paperpile.com/b/SZqQGw/0CBjJ)

[Leontiev, Oleg, and Richard B. Buxton. 2007. “Reproducibility of BOLD, Perfusion, and CMRO2 Measurements with Calibrated-BOLD fMRI.” *NeuroImage* 35 (1): 175–184.](http://paperpile.com/b/SZqQGw/xSWJP)

[Livingstone, Margaret S., Justin L. Vincent, Michael J. Arcaro, Krishna Srihasam, Peter F. Schade, and Tristram Savage. 2017. “Development of the Macaque Face-Patch System.” *Nature Communications* 8 (1): 14897.](http://paperpile.com/b/SZqQGw/ToPTe)

[Livingstone, M., and D. Hubel. 1988. “Segregation of Form, Color, Movement, and Depth: Anatomy, Physiology, and Perception.” *Science (New York, N.Y.)* 240 (4853): 740–749.](http://paperpile.com/b/SZqQGw/zz3wC)

[Margulies, Daniel S., Satrajit S. Ghosh, Alexandros Goulas, et al. 2016. “Situating the Default-Mode Network along a Principal Gradient of Macroscale Cortical Organization.” *Proceedings of the National Academy of Sciences of the United States of America* 113 (44): 12574–12579.](http://paperpile.com/b/SZqQGw/hVYS)

[Nasr, Shahin, Ning Liu, Kathryn J. Devaney, et al. 2011. “Scene-Selective Cortical Regions in Human and Nonhuman Primates.” *The Journal of Neuroscience: The Official Journal of the Society for Neuroscience* 31 (39): 13771–13785.](http://paperpile.com/b/SZqQGw/2kEuJ)

[Nie, Feng, and Margaret Wong-Riley. 1999. “Nuclear Respiratory Factor-2 Subunit Protein: Correlation with Cytochrome Oxydase and Regulation by Functional Activity in the Monkey Primary Visual Cortex.” *The Journal of Comparative Neurology*, Brain workCerebral cort, vol. 404 (3): 310–320.](http://paperpile.com/b/SZqQGw/hBpYK)

[Oishi, Hiroki, Vladimir K. Berezovskii, Margaret S. Livingstone, Kevin S. Weiner, and Michael J. Arcaro. 2024. “Inferotemporal Face Patches Are Histo-Architectonically Distinct.” *Cell Reports* 43 (9): 114732.](http://paperpile.com/b/SZqQGw/VaHuV)

[Perkins, Guy A., Mark H. Ellisman, and Donald A. Fox. 2003. “Three-Dimensional Analysis of Mouse Rod and Cone Mitochondrial Cristae Architecture: Bioenergetic and Functional Implications.” *Molecular Vision* 9 (March): 60–73.](http://paperpile.com/b/SZqQGw/arHQb)

[Peterson, Matthew F., Ian Zaun, Harris Hoke, Guo Jiahui, Brad Duchaine, and Nancy Kanwisher. 2019. “Eye Movements and Retinotopic Tuning in Developmental Prosopagnosia.” *Journal of Vision* 19 (9): 7.](http://paperpile.com/b/SZqQGw/LDTcM)

[Rączy, Katarzyna, Madita Linke, Job van den Hurk, et al. 2025. “Visual and Auditory Object Representations in Ventral Visual Cortex after Restoring Sight in Humans.” *Human Brain Mapping* 46 (12). https://doi.org/](http://paperpile.com/b/SZqQGw/slBAr)[10.1002/hbm.70316](http://dx.doi.org/10.1002/hbm.70316)[.](http://paperpile.com/b/SZqQGw/slBAr)

[Rima, Samy, Benoit R. Cottereau, Yseut Héjja-Brichard, Yves Trotter, and Jean-Baptiste Durand. 2020. “Wide-Field Retinotopy Reveals a New Visuotopic Cluster in Macaque Posterior Parietal Cortex.” *Brain Structure & Function* 225 (8): 2447–2461.](http://paperpile.com/b/SZqQGw/TamUA)

[Silverman, M. S., and R. B. Tootell. 1987. “Modified Technique for Cytochrome Oxidase Histochemistry: Increased Staining Intensity and Compatibility with 2-Deoxyglucose Autoradiography.” *Journal of Neuroscience Methods* 19 (1): 1–10.](http://paperpile.com/b/SZqQGw/9kPfZ)

[Srihasam, Krishna, Justin L. Vincent, and Margaret S. Livingstone. 2014. “Novel Domain Formation Reveals Proto-Architecture in Inferotemporal Cortex.” *Nature Neuroscience* 17 (12): 1776–1783.](http://paperpile.com/b/SZqQGw/S8TdC)

[Stehr, Daniel A., Yiyuan Zhang, Anusha Patgiri, Alexis Kidder, Kendrick Kay, and Bradley Duchaine. 2025. “Developmental Prosopagnosics Have Normal Spatial Integration in Posterior Ventral Face-Selective Regions.” In *eLife*. ELife, November 21. https://doi.org/](http://paperpile.com/b/SZqQGw/uaZRR)[10.7554/elife.108657.1](http://dx.doi.org/10.7554/elife.108657.1)[.](http://paperpile.com/b/SZqQGw/uaZRR)

[Stigliani, Anthony, Brianna Jeska, and Kalanit Grill-Spector. 2017. “Encoding Model of Temporal Processing in Human Visual Cortex.” *Proceedings of the National Academy of Sciences of the United States of America* 114 (51): E11047–E11056.](http://paperpile.com/b/SZqQGw/KzhoT)

[Tootell, R. B., N. K. Hadjikhani, W. Vanduffel, et al. 1998. “Functional Analysis of Primary Visual Cortex (V1) in Humans.” *Proceedings of the National Academy of Sciences of the United States of America* 95 (3): 811–817.](http://paperpile.com/b/SZqQGw/FALbV)

[Vogel, Jacob W., Aaron F. Alexander-Bloch, Konrad Wagstyl, et al. 2024. “Deciphering the Functional Specialization of Whole-Brain Spatiomolecular Gradients in the Adult Brain.” *Proceedings of the National Academy of Sciences of the United States of America* 121 (25): e2219137121.](http://paperpile.com/b/SZqQGw/tFscw)

[Wandell, Brian A., Serge O. Dumoulin, and Alyssa A. Brewer. 2007. “Visual Field Maps in Human Cortex.” *Neuron* 56 (2): 366–383.](http://paperpile.com/b/SZqQGw/U0EEq)

[Weber, Bruno, Anna Lena Keller, Johannes Reichold, and Nikos K. Logothetis. 2008. “The Microvascular System of the Striate and Extrastriate Visual Cortex of the Macaque.” *Cerebral Cortex (New York, N.Y.: 1991)* 18 (10): 2318–2330.](http://paperpile.com/b/SZqQGw/61ppz)

[Wong-Riley, M. T. 1989. “Cytochrome Oxidase: An Endogenous Metabolic Marker for Neuronal Activity.” *Trends in Neurosciences* 12 (3): 94–101.](http://paperpile.com/b/SZqQGw/axseq)

[Wong-Riley, M. T., and C. Welt. 1980. “Histochemical Changes in Cytochrome Oxidase of Cortical Barrels after Vibrissal Removal in Neonatal and Adult Mice.” *Proceedings of the National Academy of Sciences of the United States of America* 77 (4): 2333–2337.](http://paperpile.com/b/SZqQGw/3YPQH)

[Yeo, B. T. Thomas, Fenna M. Krienen, Jorge Sepulcre, et al. 2011. “The Organization of the Human Cerebral Cortex Estimated by Intrinsic Functional Connectivity.” *Journal of Neurophysiology* 106 (3): 1125–1165.](http://paperpile.com/b/SZqQGw/ckgYP)
