# Supplementary material for: Metabolic organization of macaque visual cortex reflects visual field topography and perceptual specialization
Source: PLoS Biol. 2026 Jun 8;24(6):e3003847. doi: 10.1371/journal.pbio.3003847 (PMC13286273; doi:10.1371/journal.pbio.3003847)
Supplement: Peer review history

# Editor report: Decision Letter 0

**DOI:** 10.1371/journal.pbio.3003847.r001

**Authors:** Taylor Hart, PhD

24 Oct 2025

Dear Dr Oishi,

Thank you for submitting your manuscript entitled "Metabolic organization of macaque visual cortex reflects retinotopic eccentricity and category selectivity" for consideration as a Short Report by PLOS Biology.

Your manuscript has now been evaluated by the PLOS Biology editorial staff, as well as by an academic editor with relevant expertise, and I am writing to let you know that we would like to send your submission out for external peer review.

However, before we can send your manuscript to reviewers, we need you to complete your submission by providing the metadata that is required for full assessment. To this end, please login to Editorial Manager where you will find the paper in the ‘Submissions Needing Revisions’ folder on your homepage. Please click ‘Revise Submission’ from the Action Links and complete all additional questions in the submission questionnaire.

Once your full submission is complete, your paper will undergo a series of checks in preparation for peer review. After your manuscript has passed the checks it will be sent out for review. To provide the metadata for your submission, please Login to Editorial Manager (https://www.editorialmanager.com/pbiology) within two working days, i.e. by Oct 26 2025 11:59PM.

If your manuscript has been previously peer-reviewed at another journal, PLOS Biology is willing to work with those reviews in order to avoid re-starting the process. Submission of the previous reviews is entirely optional and our ability to use them effectively will depend on the willingness of the previous journal to confirm the content of the reports and share the reviewer identities. Please note that we reserve the right to invite additional reviewers if we consider that additional/independent reviewers are needed, although we aim to avoid this as far as possible. In our experience, working with previous reviews does save time.

If you would like us to consider previous reviewer reports, please edit your cover letter to let us know and include the name of the journal where the work was previously considered and the manuscript ID it was given. In addition, please upload a response to the reviews as a ‘Prior Peer Review’ file type, which should include the reports in full and a point-by-point reply detailing how you have or plan to address the reviewers’ concerns.

During the process of completing your manuscript submission, you will be invited to opt-in to posting your pre-review manuscript as a bioRxiv preprint. Visit http://journals.plos.org/plosbiology/s/preprints for full details. If you consent to posting your current manuscript as a preprint, please upload a single Preprint PDF.

Feel free to email us at plosbiology@plos.org if you have any queries relating to your submission.

Kind regards,

Taylor

Taylor Hart, PhD,

Associate Editor

PLOS Biology

thart@plos.org

# Peer review: Decision Letter 1

**DOI:** 10.1371/journal.pbio.3003847.r002

**Authors:** Taylor Hart, PhD

15 Dec 2025

Dear Dr Oishi,

Thank you for your patience while your manuscript "Metabolic organization of macaque visual cortex reflects retinotopic eccentricity and category selectivity" was peer-reviewed at PLOS Biology. It has now been evaluated by the PLOS Biology editors, an Academic Editor with relevant expertise, and by several independent reviewers.

In light of the reviews, which you will find at the end of this email, we would like to invite you to revise the work to thoroughly address the reviewers’ reports.

As you will see, the reviewers expressed interest in the findings. However, they also noted several areas where the insights from the study could be deepened through examining corroborative data or providing additional analyses. In addition, they pointed out the need for more documentation, further contextualization within the existing literature, and an expanded Discussion section. In your revision, you should address the reviewers’ requests for further analyses, where feasible, and provide a thorough response to all of their comments.

Given the extent of revision needed, we cannot make a decision about publication until we have seen the revised manuscript and your response to the reviewers’ comments. Your revised manuscript is likely to be sent for further evaluation by all or a subset of the reviewers.

In addition to these revisions, you will need to complete some formatting changes, which you will receive in a follow up email. A member of our team will be in touch with a set of requests shortly.

We expect to receive your revised manuscript within 3 months. Please email us (plosbiology@plos.org) if you have any questions or concerns, or would like to request an extension.

At this stage, your manuscript remains formally under active consideration at our journal; please notify us by email if you do not intend to submit a revision so that we may withdraw it.

**IMPORTANT - SUBMITTING YOUR REVISION**

Your revisions should address the specific points made by each reviewer. Please submit the following files along with your revised manuscript:

1. A ‘Response to Reviewers’ file - this should detail your responses to the editorial requests, present a point-by-point response to all of the reviewers’ comments, and indicate the changes made to the manuscript.

*NOTE: In your point-by-point response to the reviewers, please provide the full context of each review. Do not selectively quote paragraphs or sentences to reply to. The entire set of reviewer comments should be present in full and each specific point should be responded to individually, point by point.

You should also cite any additional relevant literature that has been published since the original submission and mention any additional citations in your response.

2. In addition to a clean copy of the manuscript, please also upload a ‘track-changes’ version of your manuscript that specifies the edits made. This should be uploaded as a "Revised Article with Changes Highlighted" file type.

*Re-submission Checklist*

When you are ready to resubmit your revised manuscript, please refer to this re-submission checklist: https://plos.io/Biology_Checklist

To submit a revised version of your manuscript, please go to https://www.editorialmanager.com/pbiology/ and log in as an Author. Click the link labelled ‘Submissions Needing Revision’ where you will find your submission record.

Please make sure to read the following important policies and guidelines while preparing your revision:

*Published Peer Review*

Please note while forming your response, if your article is accepted, you may have the opportunity to make the peer review history publicly available. The record will include editor decision letters (with reviews) and your responses to reviewer comments. If eligible, we will contact you to opt in or out. Please see here for more details:

https://blogs.plos.org/plos/2019/05/plos-journals-now-open-for-published-peer-review/

*PLOS Data Policy*

Please note that as a condition of publication PLOS’ data policy (http://journals.plos.org/plosbiology/s/data-availability) requires that you make available all data used to draw the conclusions arrived at in your manuscript. If you have not already done so, you must include any data used in your manuscript either in appropriate repositories, within the body of the manuscript, or as supporting information (N.B. this includes any numerical values that were used to generate graphs, histograms etc.). For an example see here: http://www.plosbiology.org/article/info%3Adoi%2F10.1371%2Fjournal.pbio.1001908#s5

*Blot and Gel Data Policy*

We require the original, uncropped and minimally adjusted images supporting all blot and gel results reported in an article’s figures or Supporting Information files. We will require these files before a manuscript can be accepted so please prepare them now, if you have not already uploaded them. Please carefully read our guidelines for how to prepare and upload this data: https://journals.plos.org/plosbiology/s/figures#loc-blot-and-gel-reporting-requirements

*Protocols deposition*

To enhance the reproducibility of your results, we recommend that if applicable you deposit your laboratory protocols in protocols.io, where a protocol can be assigned its own identifier (DOI) such that it can be cited independently in the future. Additionally, PLOS ONE offers an option for publishing peer-reviewed Lab Protocol articles, which describe protocols hosted on protocols.io. Read more information on sharing protocols at https://plos.org/protocols?utm_medium=editorial-email&utm_source=authorletters&utm_campaign=protocols

Thank you again for your submission to our journal. We hope that our editorial process has been constructive thus far, and we welcome your feedback at any time. Please don’t hesitate to contact us if you have any questions or comments.

Sincerely,

Taylor

Taylor Hart, PhD,

Associate Editor

PLOS Biology

thart@plos.org

------------------------------------

REVIEWS:

Reviewer #1: A main point of the present manuscript is that face-selective region ML (biased toward central vision) exhibits higher cytochrome oxidase (CO) intensity than the scene-selective region LPP (biased toward peripheral vision). The Authors propose this difference is due to a close association of energy consumption with retinotopic eccentricity. They generalize as a second major point, that cortical metabolic resources are shaped by processing demands of visual perception. This is an interesting proposal, and the Authors are experienced and well positioned to address this technically and conceptually, by virtue of their several closely related previous publications. There are several points, however, for further clarification or improvement.

1. The comparison of CO intensity and especially of depthwise differences are under documented; the textual description does not provide detail as to sublayers: "middle layers" are layer 4? or as in the literature, deep layer 3 and upper layer4 ? Or possible fluctuations along the tangential domain. Is there evidence of subdivisions within one or both areas? A figure would be helpful, similar to 2D in Arcaro et al., 2024 but at higher magnification.

Why is the depth dimension even introduced? "These findings demonstrate that face- and scene-selective regions...differ in metabolic staining intensity, and that this difference spans the entire cortical depth."

2. Results Section 2 (CO intensity correlates with eccentricity.....). The proposed link of CO intensity to retinotopic organization is, as stated, rather speculative (despite last sentence in that section). One problem is the absence of physiological mapping in the same animal. The areas in these two animals are localized by comparison with previous studies and the CO pattern. Another problem is the possible confound of multimodality. Finally, another problem becomes the heavy reliance on CO as a proxy for metabolic levels. There is a huge literature on category-selective patches (face or scene). Are there further corroborative data supportive of specifically metabolic differences; for example, cellular density? vascularity? receptor distribution? That would contribute fruther support, although perhaps something for the Discussion.

3. As stated in the Discussion, the proposed relationship between eccentricity and CO intensity "may be shaped by multiple co-aligned factors." Instead of the rather general discussion that follows, a stronger argument might be to review evidence about CO and metabolism, including corroborative evidence from vascularity or (as mentioned above, receptor distribution? gene expression?) This is insufficiently reviewed in the present manuscript, even as relates to the basics of CO pre- or postsynaptic influence.

4) There is an extensive literature, especially for human brain, in relation to gradient-wise organization (J.W. Vogel et al., 2024 and others). In my opinion, it is important for the Authors to briefly review this (in the context of their emphasis on "eccentricity").

Reviewer #2: Oishi et al. combined cytochrome oxidase (CO) in 2 monkeys with fMRI to assess retinotopic maps and visual category selectivity in 9 monkeys. They found that CO indicates metabolic demands which vary with eccentricity (higher for central vision), resulting in a particularly expressed gradient in ML (middle lateral face patch) compared to LPP (lateral place patch).

To the best of my knowledge, these results are new and have the potential to uncover how higher-order representations arise in the visual cortical system. The manuscript is very well written. Since I am not a monkey researcher, I am not able to judge the anatomical methods.

The only major concern I have is the scare discussion, which seems to underuse the exceptional data and results reported in the Results section, see below.

Remark: Unfortunately, the PDF for download did not have page or line numbers. Thus, when referring to page numbers, I use those of the PDF file, which starts with a questionnaire.

Page 11: ML and LPP are defined in the Results section but used, e.g., in the abstract earlier; better add definition to the abstract.

Page 12: "These findings demonstrate that cortical metabolic architecture reflects functional layout, shaped by both the spatial structure of visual input and the processing demands of distinct visual domains." Are these independent aspects? It could be argued that the spatial structure is the result of the processing demands.

Page 13: Please add how face selectivity and scene selectivity were defined statistically.

Figure 3: please indicate the different scales for M1 and M2.

Minor suggestion: Figure 4: In the text results of the deep portion of the grey matter are discussed before those of the superficial portion. I would mirror this in Figure 4 (A-left: Deep, A-right: superficial)

Page 21: The idea that visual experience contributes to metabolic organization is in accord with studies in individuals with reversed congenital cataracts, who show a lower representation of the fovea (https://doi.org/10.1016/j.cub.2023.10.010), as well as reduced face selectivity in inferior temporal cortex (https://doi.org/10.1002/hbm.70316).

Please explain how developmental prosopagnosia might help to clarify how visual experience/ category experience shapes metabolic architecture. These individuals have, to the best of my knowledge, "typical" visual input from the eyes and no V1 deficits. Sight recovery individuals with a history of congenital blindness might be a more fruitful study approach in humans.

Discussion: is a bit scant and does not yet deals with all aspects of the results nor provides mechanistic explanations. For example, it is considered that cones are more energy demanding than rods. How does this translate to cortical neurons? Are neural compositions different for different parts of the inferior temporal cortex?

The role of receptive field size is discussed. However, human fMRI studies have shown that these increase for face-selective regions in IT compared to early visual cortex; however, ML seems to be characterized by especially high CO gradients.

The results of Figure 4 are not discussed at all. Why are deviations from the average retinotopic - CO relation different for different cortical layers, and why does this interact with ROI (e.g., V1 vs. ML)? This finding seems to be crucial and might have the potential to make a significant contribution to our understanding of how higher-order representations emerge in the visual system.

Nice prospects are given for the dorsal stream, but any concrete hypothesis? The discussion is a bit decoupled from the abstract and main conclusion. I might have missed direct comparisons between regions, e.g., between ML and LPP.

Questions: Were the 2 monkeys on which CO histology was performed among the monkeys for which fMRI retinotopy and categorical organization in the inferior temporal cortex were assessed? I would add an explicit statement to the text.

Reviewer #3: This is an interesting manuscript that examined the relationship between fMRI measures of eccentricity with cytochrome oxidase staining as an indicator of metabolism. The presentation is concise and in general the study is very well presented and written. A general overview of previous work on this area on primary sensory systems is likely necessary to catch up the reviewer on details of cytochrome oxidase and in vivo metabolism measurements. As is, the work reads like this is a new study without appropriate recognition of prior work (2DG results and/or other imaging results from PET or MRI would be very helpful). This work could benefit from short-reaching extensions, even if brief, like comparing the CO staining with depth with mapping signals of eccentricity (amplitude) even if those are biased to the superficial layers. Another possibility is discussing the relationship between CO staining and vascular or capillary density as these are likely linked. Other than that, I agree that evaluating metabolic markers with function remains an important understudied aspect in brain imaging research.

# Author comment: Author response to Decision Letter 2

**DOI:** 10.1371/journal.pbio.3003847.r003

13 Apr 2026

Attachment Submitted filename: Response to reviewers_PLOSBiology_Oishi_submission.docx

# Peer review: Decision Letter 2

**DOI:** 10.1371/journal.pbio.3003847.r004

**Authors:** Taylor Hart, PhD

19 May 2026

Dear Dr Oishi,

Thank you for your patience while we considered your revised manuscript "Metabolic organization of macaque visual cortex reflects retinotopic eccentricity and category selectivity" for publication as a Short Report at PLOS Biology. This revised version of your manuscript has been evaluated by the PLOS Biology editors, the Academic Editor, and the original reviewers.

Based on the reviews, we are likely to accept this manuscript for publication. Please also make sure to address the following data and other policy-related requests.

IMPORTANT: Please ensure that your next revision addresses all of the following points:

-----------------

**Title:

We worry that the term ‘retinotopic eccentricity’ may not be accessible to the journal’s broad readership, so we have suggested an alternative option that is a bit more general. Is this, or something similar, acceptable to you?

"Metabolic organization of macaque visual cortex reflects processing demands of visual perception"

**Ethics:

Please begin the Materials and Methods section with a separate sub-heading titled ‘Ethics Statement’, and include all relevant protocol/permit/project/approval numbers.

**Data:

Thank you for uploading the data and code to OSF. Please select a License for the repository, and include a README. Please also confirm that all of the numerical data underlying the following figure panels are included in the repository:

1D

4B

S3AB

Please cite the location of the data clearly in all relevant main and supplementary Figure legends, e.g. “The data underlying this Figure can be found in https://doi.org/10.5281/zenodo.XXXXX”

Please also confirm that your Data Location Statement accurately reflects where all of the data can be found.

-----------------

As you address these items, please take this last chance to review your reference list to ensure that it is complete and correct. If you have cited papers that have been retracted, please include the rationale for doing so in the manuscript text, or remove these references and replace them with relevant current references. Any changes to the reference list should be mentioned in the cover letter that accompanies your revised manuscript.

In addition to these revisions, you may need to complete some formatting changes, which you will receive in a follow up email. A member of our team will be in touch with a set of requests shortly. If you do not receive a separate email within a few days, please assume that checks have been completed, and no additional changes are required.

We expect to receive your revised manuscript within two weeks.

To submit your revision, please go to https://www.editorialmanager.com/pbiology/ and log in as an Author. Click the link labelled ‘Submissions Needing Revision’ to find your submission record. Your revised submission must include the following:

- a cover letter that should detail your responses to any editorial requests, if applicable, and whether changes have been made to the reference list

- a Response to Reviewers file that provides a detailed response to the reviewers’ comments (if applicable, if not applicable please do not delete your existing ‘Response to Reviewers’ file.)

- a track-changes file indicating any changes that you have made to the manuscript.

NOTE: If Supporting Information files are included with your article, note that these are not copyedited and will be published as they are submitted. Please ensure that these files are legible and of high quality (at least 300 dpi) in an easily accessible file format. For this reason, please be aware that any references listed in an SI file will not be indexed. For more information, see our Supporting Information guidelines:

https://journals.plos.org/plosbiology/s/supporting-information

*Published Peer Review History*

Please note that you may have the opportunity to make the peer review history publicly available. The record will include editor decision letters (with reviews) and your responses to reviewer comments. If eligible, we will contact you to opt in or out. Please see here for more details:

https://plos.org/published-peer-review-history/

*Press*

Should you, your institution’s press office or the journal office choose to press release your paper, please ensure you have opted out of Early Article Posting on the submission form. We ask that you notify us as soon as possible if you or your institution is planning to press release the article.

*Protocols deposition*

To enhance the reproducibility of your results, we recommend that if applicable you deposit your laboratory protocols in protocols.io, where a protocol can be assigned its own identifier (DOI) such that it can be cited independently in the future. Additionally, PLOS ONE offers an option for publishing peer-reviewed Lab Protocol articles, which describe protocols hosted on protocols.io. Read more information on sharing protocols at https://plos.org/protocols?utm_medium=editorial-email&utm_source=authorletters&utm_campaign=protocols

Please do not hesitate to contact me should you have any questions.

Sincerely,

Taylor

Taylor Hart, PhD,

Associate Editor

thart@plos.org

PLOS Biology

REVIEWS

Reviewer #1: I’ve reviewed the revisions and Author comments to my review, as well as, more briefly, the extensive comments to the other reviewers. The Authors have obviously done an acceptable and conscientious job in replies and revisions. The paper leans on the very complex issue of "metabolism," which is clearly worthy of a more full-scale scrutiny. However, in my opinion, the revised manuscript fully meets current professional standards, and will be a solid addition to the literature.

Reviewer #2: I am impressed how detailed the authors responded to comments and how carefully they revised the manuscript. This is an outstanding publication! I do not have any additional comments.

Reviewer #3: All my comments were satisfactorily addressed.

# Author comment: Author response to Decision Letter 3

**DOI:** 10.1371/journal.pbio.3003847.r005

26 May 2026

Attachment Submitted filename: Response_to_reviewers_PLOSBiology_Oishi_submission_auresp_3.docx

# Editor report: Decision Letter 3

**DOI:** 10.1371/journal.pbio.3003847.r006

**Authors:** Taylor Hart, PhD

27 May 2026

Dear Dr Oishi,

Thank you for the submission of your revised Short Report "Metabolic organization of macaque visual cortex reflects visual field topography and perceptual specialization" for publication in PLOS Biology. On behalf of my colleagues and the Academic Editor, Laura Lewis, I am pleased to say that we can in principle accept your manuscript for publication, provided you address any remaining formatting and reporting issues. These will be detailed in an email you should receive within 2-3 business days from our colleagues in the journal operations team; no action is required from you until then. Please note that we will not be able to formally accept your manuscript and schedule it for publication until you have completed any requested changes.

Please take a minute to log into Editorial Manager at http://www.editorialmanager.com/pbiology/, click the "Update My Information" link at the top of the page, and update your user information to ensure an efficient production process.

PRESS

We frequently collaborate with press offices. If your institution or institutions have a press office, please notify them about your upcoming paper at this point, to enable them to help maximise its impact. If the press office is planning to promote your findings, we would be grateful if they could coordinate with biologypress@plos.org. If you have previously opted in to the early version process, we ask that you notify us immediately of any press plans so that we may opt out on your behalf.

We also ask that you take this opportunity to read our Embargo Policy regarding the discussion, promotion and media coverage of work that is yet to be published by PLOS. As your manuscript is not yet published, it is bound by the conditions of our Embargo Policy. Please be aware that this policy is in place both to ensure that any press coverage of your article is fully substantiated and to provide a direct link between such coverage and the published work. For full details of our Embargo Policy, please visit http://www.plos.org/about/media-inquiries/embargo-policy/.

Thank you again for choosing PLOS Biology for publication and supporting Open Access publishing. We look forward to publishing your study.

Sincerely,

Taylor

Taylor Hart, PhD,

Associate Editor

PLOS Biology

thart@plos.org
